# Pathogenic T Cells in Celiac Disease Change Phenotype on Gluten Challenge: Implications for T‐Cell‐Directed Therapies

**DOI:** 10.1002/advs.202102778

**Published:** 2021-09-08

**Authors:** Asbjørn Christophersen, Stephanie Zühlke, Eivind G. Lund, Omri Snir, Shiva Dahal‐Koirala, Louise Fremgaard Risnes, Jørgen Jahnsen, Knut E. A. Lundin, Ludvig M. Sollid

**Affiliations:** ^1^ KG Jebsen Coeliac Disease Research Centre University of Oslo Oslo 0372 Norway; ^2^ Institute of Clinical Medicine University of Oslo Oslo 0450 Norway; ^3^ Department of Rheumatology Dermatology and Infectious Diseases Oslo University Hospital Oslo 0372 Norway; ^4^ Department of Immunology Oslo University Hospital Oslo 0372 Norway; ^5^ Department of Gastroenterology Akershus University Hospital Lørenskog 1478 Norway; ^6^ Department of Gastroenterology Oslo University Hospital Rikshospitalet Oslo 0372 Norway

**Keywords:** celiac disease, gluten challenge, mass cytometry, RNA‐Seq, T cells

## Abstract

Gluten‐specific CD4^+^ T cells being drivers of celiac disease (CeD) are obvious targets for immunotherapy. Little is known about how cell markers harnessed for T‐cell‐directed therapy can change with time and upon activation in CeD and other autoimmune conditions. In‐depth characterization of gluten‐specific CD4^+^ T cells and CeD‐associated (CD38^+^ and CD103^+^) CD8^+^ and *γδ* ^+^ T cells in blood of treated CeD patients undergoing a 3 day gluten challenge is reported. The phenotypic profile of gluten‐specific cells changes profoundly with gluten exposure and the cells adopt the profile of gluten‐specific cells in untreated disease (CD147^+^, CD70^+^, programmed cell death protein 1 (PD‐1)^+^, inducible T‐cell costimulator (ICOS)^+^, CD28^+^, CD95^+^, CD38^+^, and CD161^+^), yet with some markers being unique for day 6 cells (C‐X‐C chemokine receptor type 6 (CXCR6), CD132, and CD147) and with integrin *α*4*β*7, C‐C motif chemokine receptor 9 (CCR9), and CXCR3 being expressed stably at baseline and day 6. Among gluten‐specific CD4^+^ T cells, 52% are CXCR5^+^ at baseline, perhaps indicative of germinal‐center reactions, while on day 6 all are CXCR5^−^. Strikingly, the phenotypic profile of gluten‐specific CD4^+^ T cells on day 6 largely overlaps with that of CeD‐associated (CD38^+^ and CD103^+^) CD8^+^ and *γδ* ^+^ T cells. The antigen‐induced shift in phenotype of CD4^+^ T cells being shared with other disease‐associated T cells is relevant for development of T‐cell‐directed therapies.

## Graphical abstract

Celiac disease is driven by CD4^+^ T cells specific to gluten. Gluten exposure induces expression of potential therapeutic target molecules by the cells. Many newly expressed markers are shared with celiac disease‐associated CD8^+^ and *γδ* ^+^ T cells being activated (CD38^+^) and gut‐homing (CD103^+^). The findings are relevant to T‐cell‐directed therapy of celiac disease and other T‐cell driven autoimmune conditions.

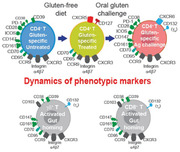

## 1 Introduction

Celiac disease (CeD) is a prevalent autoimmune‐like condition which is caused by an immune response to cereal gluten proteins.^[^ ^1^ ^]^ The disease is treated with a gluten‐free diet. When CeD patients eliminate gluten from their diet, disease‐related symptoms disappear and the lesion of the upper small intestine normalizes. The disease has a strong genetic basis and human leukocyte antigen (HLA) is the chief genetic determinant.^[^ ^2^ ^]^ The patients uniquely possess gluten‐specific CD4^+^ T cells that recognize deamidated gluten peptide bound to the disease‐associated HLA variants HLA‐DQ2.5, HLA‐DQ2.2, or HLA‐DQ8. These gluten‐specific CD4^+^ T cells can be considered drivers of the disease pathology due to their cytokines and phenotypic profile, HLA‐restriction and presence in the lamina propria of the inflamed gut tissue.^[^ ^3^ ^]^ Detection of such pathogenic, gluten‐specific T cells can be done with ELISPOT assay^[^ ^4^ ^]^ or with tetramerized HLA‐DQ2.5, HLA‐DQ2.2, or HLA‐DQ8 molecules loaded with their respective gluten peptide ligands.^[^ ^5^, ^6^ ^]^ Harnessing HLA‐DQ:gluten tetramer staining in combination with T‐cell receptor sequencing, we previously demonstrated that gluten‐specific T‐cell clonotypes overlap between gut and blood in CeD patients.^[^ ^7^ ^]^ Further, by using HLA‐DQ:gluten tetramers in combination with mass cytometry that allows for multiparametric phenotyping, we demonstrated that the cells have a narrow phenotype.^[^ ^8^ ^]^ This phenotype was similar for cells in blood and gut and included multiple features typical of CD4^+^ T cells that provide help to B cells forantibody production.^[^ ^8^ ^]^

In addition to the gluten‐specific CD4^+^ T cells in lamina propria, the celiac gut lesion is characterized by an increased number of intraepithelial CD8^+^ and *γδ* ^+^ T cells.^[^ ^3^ ^]^ While the intraepithelial CD8^+^ T cells seem to be directly responsible for the killing of the epithelial cells that cover the intestinal lumen,^[^ ^9^ ^]^ the function of the *γδ* ^+^ T cells is more elusive.

When treated patients are orally challenged with gluten, there is a wave of gluten‐specific CD4^+^T cells in peripheral blood 6–8 days after the initiation of the challenge.^[^ ^4^, ^5^, ^10^ ^]^ Such cells, which are not present in healthy subjects,^[^ ^5^, ^11^ ^]^ are present in the treated patients also before the gluten challenge, but at lower frequency.^[^ ^7^ ^]^ Together, these findings of persistent and re‐expanding antigen‐specific T cells help to explain why CeD is a chronic disorder and why gluten‐specific CD4^+^ T cells are attractive targets for novel T‐cell‐directed therapies.^[^ ^12^ ^]^ However, in parallel to the increase in gluten‐specific CD4^+^ T cells, there is a gluten‐challenge‐induced surge of activated (CD38^+^), gut‐homing (CD103^+^) CD8^+^ and *γδ*+ T cells in peripheral blood on day 6.^[^ ^13^, ^14^ ^]^ We recently demonstrated that clonotypes of such CD8^+^ and *γδ* ^+^ T cells in blood are shared between cells of the intraepithelial compartment.^[^ ^15^ ^]^ Unlike the gluten‐specific CD4^+^ T cells, however, the antigen specificities of these circulating, CeD‐associated CD38^+^ CD103^+^ CD8^+^ and *γδ* ^+^ T cells, are unknown. Obviously, further characterization of these CD8^+^ and *γδ* ^+^ T cells is needed, especially as they may prove to be important targets in a therapeutic setting.

Here, we have used a cocktail of five distinct HLA‐DQ2.5:gluten tetramers (hereafter named tetramer) representing immunodominant T‐cell epitopes and isolated gluten‐specific cells in HLA‐DQ2.5‐positive patients in remission at baseline and at day 6 (d6) after a 3‐day gluten challenge. Bulk populations of Tetramer^+^ and Tetramer^−^ cells at d6 were subjected to RNA‐sequencing (RNA‐seq), and differentially expressed genes were identified. Based on the results, we identified cell surface markers that are likely to differ gluten‐specific cells from other circulating CD4^+^ T cells and, which may show dynamic range upon gluten exposure. Suitable antibodies were identified and tested along with HLA‐DQ2.5:gluten tetramers by mass cytometry. In addition, we used the antibody panel to characterize CD38^+^ CD103^+^ CD8^+^ and *γδ* ^+^ T cells appearing concomitantly with the gluten‐specific CD4^+^ T cells in blood after oral gluten challenge. Overall, the approach allowed for a comprehensive analysis of changes in marker expression on gluten‐specific CD4^+^ T cells upon gluten challenge, and the results were contrasted with marker expression in gluten‐specific blood T cells of untreated CeD patients, i.e., patients consuming gluten, and with that of the CeD associated (CD38^+^ CD103^+^) CD8^+^ and *γδ* ^+^ T cells. The results give a picture of dynamic changes in the phenotype of the gluten‐specific T cells upon exposure to gluten. Further, the work identifies markers both being useful for cell monitoring as well as being potential targets for therapies aiming at T cells.

## 2 Results

### 2.1 Antigen‐Induced Increase in Gluten‐Specific Blood T Cells

**Figure 1:**
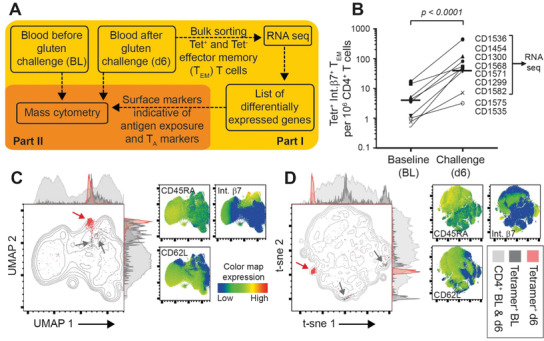
Study design and initial clustering of gluten HLA‐DQ2.5:gluten tetramer‐binding (Tet^+^) CD4^+^ T cells. A) Depiction of study design. In part I, gut homing (integrin *β*7^+^) Tet^+^ and Tet^−^ effector memory T (T_EM_) (CD45RA^−^, CD62L^−^) cells were bulk‐sorted and analyzed with RNA‐seq (*n* = 6). In Part II, mass cytometry was performed (*n* = 10). Based on surface markers previously reported to characterize gluten‐specific CD4^+^ T cells in untreated celiac disease (CeD), i.e., T‐cell autoimmune (T_A_) markers, and with addition of surface markers identified from RNA‐seq results, a mass cytometry staining panel was designed. Labeled antibodies were used to stain PBMCs obtained before (baseline, BL) and 6 days (d6) after a 3 day gluten challenge. B) Increase in numbers of Tet^+^ integrin *β*7^+^ cells per million CD4^+^ T cells (paired *t*‐test, median value indicated) as detected with flow cytometry. Patients chosen for RNA‐seq analysis are indicated. C,D) Graph plots of high‐dimensional analysis (UMAP and t‐sne) with adjunct histograms depicting all CD4^+^ T cells and visualizing Tet^+^ cells on d6 (red), baseline (dark gray) and all CD4^+^ T cells merged from baseline and d6 (light gray). Red and dark gray arrows indicate clusters of Tet^+^ cells on d6 and BL, respectively. Color maps indicate the distribution of cells stained for CD45RA, CD62L and integrin *β*7^+^ confirming location of Tet^+^ cells on baseline and d6 as these markers were used to sort cells for RNA‐seq.

Despite sparse symptoms from the oral gluten challenge (Figure S1, Supporting Information; Study Design in **Figure** **1**A), we observed an increase in gluten‐specific CD4^+^ blood T cells on day 6 in all participants, as detected by Tetramers (participant variables in Table S1 in the Supporting Information). In line with previous reports, the Tetramer^+^ cells were gut‐homing (integrin *β*7^+^) effector memory T (T_EM_, i.e., CD45RA^−^, CD62L^−^) cells,^[^ ^5^, ^16^ ^]^ and the median numbers increased from 4 to 53 per million CD4^+^ cells (Figure 1B). The flow cytometry gating strategy used is shown in Figure S2A (Supporting Information). As previously reported,^[^ ^10^, ^17^ ^]^ CD38 expression increased significantly (*p* < 0.01) on the Tetramer^+^ integrin *β*7^+^ T_EM_ cells from median 0% to 93% (Figure S2B, Supporting Information).

### 2.2 Distinct Populations of Gluten‐Specific CD4^+^ T Cells before and after Gluten Challenge

To identify markers that are unique for antigen‐specific exposure in vivo while avoiding those reflecting T‐cell memory or gut‐homing only, we undertook RNA‐seq of integrin *β*7^+^/CD4^+^/T_EM_ cells and compared the results of bulk‐sorted Tetramer^+^ and Tetramer^−^ cells from d6 samples (*n* = 6 gluten‐challenged participants). The log_2_ fold changes (FC) revealed 3178 differentially expressed (DE) genes. From these DE genes, we identified 280 genes coding cell‐surface expressed proteins (Table S2, Supporting Information).^[^ ^18^ ^]^ We selected 15 markers for which commercial antibodies are available from the total 280 markers, and designed a mass cytometry panel that also contained 16 markers used to characterize gluten‐specific CD4^+^ T cells in untreated CeD^[^ ^8^ ^]^ (Study Design in Figure 1A; staining panel in Table S3 in the Supporting Information).

High‐dimensional UMAP and t‐sne plots of mass cytometry‐derived CD4^+^ T cells from 10 gluten challenged CeD patients confirmed that the Tetramer^+^ cells at baseline and d6 cluster within CD45RA^−^, CD62L^−^, integrin *β*7^+^ cells (Figure 1C,D; participants variables in Table S4 in the Supporting Information). Surprisingly, the Tetramer^+^ cells at baseline located mainly in two clusters. These clusters were better distinguished with t‐sne in which cluster distancing is less stringent than with UMAP analysis.^[^ ^19^ ^]^

**Figure 2:**
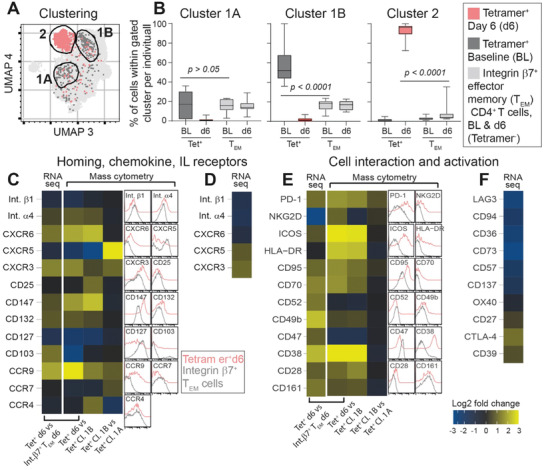
Phenotype of HLA‐DQ2.5:gluten tetramer‐binding (Tet^+^) cells before and after oral gluten challenge. A) Mass cytometry‐derived UMAP plot of integrin *β*7^+^ effector memory T (T_EM_) cells of challenge patients (*n* = 10). Cluster 1A and cluster 1B define Tet^+^ at baseline (dark gray) and cluster 2 define Tet^+^ cells on d6 (red). Light gray represents integrin *β*7^+^ T_EM_ cells merged from baseline and day 6. B) Percentage of Tet^+^ and integrin *β*7^+^ T_EM_ cells on baseline (BL) and day 6 (d6) which locate within clusters 1A, 1B, and 2, respectively. The 28 markers used to create the plots are visualized with their cellular distributions in Figure S3 (Supporting Information). C–F) Heat map depicting log_2_ fold change (FC) of homing markers, chemokine and interleukin (IL) receptors as analyzed with RNA‐seq and mass cytometry. Tet^+^ cells at d6, Tet^+^ cells at BL in clusters 1A and 1B are compared with integrin *β*7^+^ T_EM_ cells d6. To the right of the heat maps, absolute staining intensities for each individual marker as found with mass cytometry staining of Tet^+^ cells d6 and integrin *β*7^+^ T_EM_ cells are shown. In panels (D) and (F), log_2_ FC RNA expression of some additional differentially expressed homing markers not analyzed by mass cytometry are shown.

To better compare the mass cytometry and RNA‐seq data, we focused the further mass cytometry data analysis on the integrin *β*7^+^ CD4^+^ T_EM_ cells (gated from the Tetramer^+^ and pre tetramer‐enriched samples from baseline and d6, respectively), using the additional 28 T‐cell marker mass cytometry staining panel. In line with the analysis of all CD4^+^ T cells (Figure 1C,D), we identified three clusters of Tetramer^+^ cells (marker distribution in Figure S3 in the Supporting Information). Clusters 1A and 1B accommodated Tetramer^+^ cells at baseline and cluster 2 accommodated the Tetramer^+^ cells on d6 (**Figure** **2**A). At BL, significantly more Tetramer^+^ than gut‐homing T_EM_ cells located within cluster 1B (*p* < 0.00001; median 52% vs 18%), while there was no significant difference for cluster 1A. On the other hand, the Tetramer^+^ cells on d6 located almost solely within cluster 2 (median 96%), while very few d6 Tetramer^+^ cells (median <1) located within clusters 1A and 1B. Thus, cluster 2 identified a small T‐cell subset holding literally all the CD4^+^ T cells that respond specifically to gluten on challenge in vivo. On the other hand, cluster 1B defined a subset that even in absence of gluten exposure was enriched by gluten‐specific CD4^+^ T cells.

### 2.3 Phenotypic Properties of Gluten‐Specific CD4^+^ T Cells Following Oral Gluten Challenge

The combined RNA‐seq and mass cytometry analysis pointed to several novel markers of gluten‐specific cells that were induced or noticeably increased by the gluten challenge (Figure 2C–F). The mass cytometry‐derived data further detailed the properties of the two clusters of Tetramer^+^ at baseline (further comparisons for clusters 1A and 1B in Figure S4 in the Supporting Information). Tetramer^+^ on d6 expressed increased levels of C‐X‐C chemokine receptor type 6 (CXCR6) (i.e., the receptor for CXCL16), CD132 (the common *γ*‐chain) and CD147 (Figure 2A), compared with Tetramer^+^ baseline cells (clusters 1A and 1B) and gut‐homing T_EM_ cells in general. In addition, both techniques showed that d6 Tetramer^+^ cells expressed increased levels of the activation markers CD95 (FAS) and CD70 (Figure 2C). However, these two activation markers were increased also on Tetramer^+^ baseline cells when compared to gut‐homing cells in general (Figure S4, Supporting Information), possibly indicating some activation of gluten‐specific cells at baseline. In contrast to the rapid shift in cytokine receptors and raise in activation markers, Tetramer^+^ cells had increased expression of CCR9, CXCR3 and *α*4*β*7 integrin both in cluster 1B and on day 6, while integrins not associated with homing to the gut (*β*1, *α*3, *α*5, and *α*6) had a significantly lower expression in the Tetramer^+^ cells on d6. Accordingly, while their homing properties to the small intestine are stable and independent of gluten exposure, gluten‐specific CD4^+^ T cells upregulate multiple markers on d6 of gluten challenge, most notably CXCR6, CD147, CD132 and FAS.

The expression levels as measured with RNA‐seq and mass cytometry correlated well (Figure S5, Supporting Information). The exceptions were CD103 (integrin *α*E), CD52, CD47 (upregulated on the RNA level while mass cytometry staining indicated higher expression of these markers on other gut‐homing CD4^+^ blood T_EM_ cells) and NKG2D (downregulated in RNA‐seq but upregulated in mass cytometry staining). Of note, the reduced CD47 and CD52 expression on d6 Tetramer^+^ cells despite significant increase on the RNA level could potentially reflect an increased release of the molecules in soluble form due to proteolytic cleavage or as extracellular vesicles.^[^ ^20^, ^21^ ^]^ The data also were in agreement with previous observations on Tetramer^+^ cells in gut and blood in untreated CeD and on d6 after gluten challenge,^[^ ^8^ ^]^ as the d6 Tetramer^+^ cells analyzed here were CD127^−^, C‐C motif chemokine receptor 7 (CCR7)^−^, programmed cell death protein 1 (PD‐1)^+^, inducible T‐cell costimulator (ICOS)^+^, HLA‐DR^+^, CD38^+^, CD28^+^, CD161^+^, CCR5^+^, CCR6^+^, CD73^−^, CD57^−^, CD137^−^, and cytotoxic T‐lymphocyte‐associated protein 4 (CTLA‐4)^+^. CD39, expressed on gut‐ and blood‐derived gluten‐specific cells in untreated CeD patients, was not significantly increased in the RNA‐seq data, in line with our previous mass cytometry staining of Tetramer^+^ on d6.^[^ ^8^ ^]^ This indicates that CD39 increases later and rather reflects chronic (untreated CeD) than recent (gluten challenge) activation of gluten‐specific CD4^+^ T cells in vivo, contrasting in vitro based studies on gluten‐specific CD4^+^ T cells.^[^ ^22^ ^]^

Gluten‐specific CD4^+^ T cells lack several key markers reported to define regulatory T cells,^[^ ^23^ ^]^ being CD25^−^ and CD137^−^.^[^ ^8^ ^]^ Furthermore, combined CD49b/LAG3‐expression is a hallmark of type 1 regulatory (Tr1) cells that produce IL‐10 and TGF‐*β*.^[^ ^24^ ^]^ CD49b (integrin *α*2) was elevated on d6 Tetramer^+^ cells and on Tetramer^+^ cells within cluster 1B (Figure 2E), while d6 Tetramer^+^ cells had significantly lower RNA expression level of LAG3 (Figure 2F), and no shift in LAG3 staining. Thus, the great majority of gluten‐specific CD4^+^ T cells activated with an oral gluten challenge are not Tr1 cells, as defined by combined LAG3/CD49b expression.

### 2.4 CXCR5 Defines Two Clusters of Gluten‐Specific Cells at Baseline

Unlike Tetramer^+^ cells in cluster 1A, Tetramer^+^ cells in cluster 1B expressed high levels of CXCR5 in addition to higher levels of PD‐1, CCR9, CXCR3, and CCR7 (Figure 2A,C; Figure S4, Supporting Information). Combined CXCR5/PD‐1/CXCR3‐expression defines germinal center‐derived circulating T follicular helper cells (cT_FH_).^[^ ^25^ ^]^ Notably, CXCR5 was the main driver of the separate clustering in 1A and 1B, as a re‐analysis with exclusion of CXCR5 made cells from these clusters locate together (Figure S6, Supporting Information). As the cells were also CCR9^+^, integrin *α*4*β*7^+^, and CXCR3^+^, these data would be compatible with the notion that there is some CXCR5‐dependent T‐B cell interaction in CeD, possibly occurring in germinal centers of secondary or tertiary lymphoid structures in the intestine.

### 2.5 Comparing Gluten‐Specific T Cells in Untreated CeD and Treated CeD before and after Gluten Challenge

**Figure 3:**
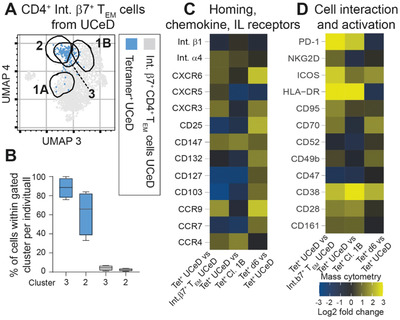
Phenotype of HLA‐DQ2.5:gluten tetramer‐binding (Tet^+^) cells of untreated CeD versus treated CeD before and after gluten challenge. A) Mass cytometry‐derived UMAP plot (generated from plot of Figure 2A) visualizing only integrin *β*7^+^ effector memory T (T_EM_) cells from untreated CeD patients. Location of cluster 3, defining most Tet^+^ cells in untreated celiac disease (UCeD) patients versus location of clusters 1A, 1B, and 2, which defined Tet^+^ cells on baseline and day 6 after gluten challenge. B) Percentage of Tet^+^ from 4 UCeD patients locating within clusters 2 and 3, respectively. The 28 markers used to create the plot are visualized with their staining distribution in Figure S3 (Supporting Information). C,D) Heat map depicting log_2_ fold change of markers analyzed by mass cytometry comparing cell types as indicated. All data include integrin *β*7^+^ CD4^+^ T_EM_ cells only.

We next included data from untreated CeD patients (participant variables in Table S5 in the Supporting Information) to assess which phenotypic markers of gluten‐specific CD4^+^ T cells, in addition to CD39,^[^ ^8^ ^]^ that can differentiate recently activated cells (of gluten challenged CeD) from chronically activated cells (of untreated CeD). Tetramer^+^ cells in blood of untreated CeD located to cluster 3, which was positioned between the majority of Tetramer^+^ at baseline, i.e., cluster 1B, and the majority of Tetramer^+^ cells on d6, i.e., cluster 2 (**Figure** **3**A). Cluster 3 confined almost all (median 95%) of the Tetramer^+^ cells in four untreated CeD patients analyzed (Figure 3B). Thus, as previously observed,^[^ ^8^ ^]^ the Tetramer^+^ cells of untreated CeD were similar to Tetramer^+^ cells on d6 for multiple markers, yet many markers with increased expression in untreated CeD had even higher expression on d6 of challenge (Figure 3C,D). Of note, CXCR6 and CD132 appeared almost unique to the Tetramer^+^ cells on d6. In addition, not all Tetramer^+^ cells on d6 had downregulated CD127 (i.e., the IL‐7 receptor), and in some challenged patients we observed higher expression levels of CD25 on Tetramer^+^ cells than in untreated CeD. Notably, some few Tetramer^+^ cells in untreated CeD expressed CXCR5, allowing these Tetramer^+^ to locate within cluster 1B. This observation supports the notion that some gluten‐specific CD4^+^ T cells may migrate to lymphoid structures in the small intestine in a CXCR5‐dependent fashion, and that this migration occurs also in active disease state.

### 2.6 Ranking Markers Defining Gluten‐Specific CD4^+^ T Cells Activated by Gluten

**Figure 4:**
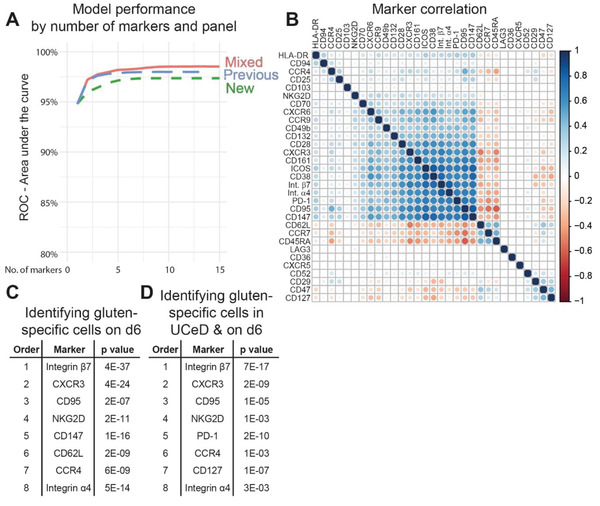
Ranking T‐cell markers defining gluten‐specific CD4^+^ T cells on day 6 after gluten challenge. A) Stepwise logistic regression analysis with receiver operating characteristics (ROC) comparing three set of markers for their ability to distinguish gluten‐specific CD4^+^ T cells from other gut‐homing (integrin *β*7^+^) effector memory (CD45RA^−^ CD62L^−^) cells. The blue line represents a previously established set of markers (previous),^[^ ^6^ ^]^ the green line represents a set of markers identified from RNA‐seq results (i.e., this study) (new) and the red line represents a mix of markers from the two sets (mixed) (comparison including also integrin *β*7, CD45RA and CD62L in Figure S6 in the Supporting Information). The predictor with the highest *p*‐value was removed at each step and model performance was evaluated using 10‐fold cross validation. B) Correlation of the 32 markers constituting the mixed panel looking at gluten tetramer binding (Tet^+^) and nontetramer binding (Tet^−^) cells 6 days after gluten challenge (d6). Samples were down‐sampled to be proportional between sample types (Tet^+/−^) and donors. C) Top 8 markers to define Tet^+^ on d6 of gluten challenge and D) for Tet^+^ cells on d6 and in untreated CeD (UCeD) combined. P values reflect the ability of each indicated marker to predict the Tet^+^ cells.

As mass cytometry involves a fairly complex cell staining protocol, we aimed to find a lower number of markers to define gluten‐specific T cells on d6 that could be applied in less demanding flow cytometry analysis. First, we asked if increasing the number of markers correlated with a more precise separation of Tetramer^+^ cells on d6 from other CD4^+^ T cells in addition to how many markers would be optimal. We stepwise trained logistic regression models on Tetramer^+^ and Tetramer^−^ integrin *β*7^+^ T_EM_ cells and compared three sets of markers, i.e., the previously established mass cytometry panel,^[^ ^8^ ^]^ the RNA‐seq derived panel and a mix of these two panels. The models were evaluated using 10‐fold cross validation and the weakest predictor was removed at each step (Table S6, Supporting Information). Interestingly, Tetramer^+^ cells on d6 were separated well from Tetramer^−^ cells with less than ten markers, using the previously established or mixed panel (**Figure** **4**A). Here, the RNA‐seq derived panel alone, performed less well. However, when including the early established^[^ ^6^ ^]^ markers to define Tetramer^+^ cells in CeD patients, i.e., CD45RA, CD62L and Integrin *β*7, the three panels performed equally well (Figure S7A, Supporting Information), confirming that these three markers are true hallmarks of gluten‐specific T cells. Remarkably, all three panels reached a plateau within 5–10 markers, indicating that many of the markers expressed by gluten‐specific T cells on d6 correlate well. Indeed, a correlation analysis on the same balanced subsample used for model training displayed a high degree of correlation for multiple markers, such as CD132, CD28, CXCR3, integrin *α*4, CD95, PD‐1, CD161, ICOS, and CD38. This observation indicates that many of the markers associated with gluten‐specific T cells are co‐expressed on individual cells—making them equally good predictors (Figure 4B). Finally, from the stepwise logistic regression model, we listed the top 8 markers from the mixed panel that define Tetramer^+^ the best on d6 (Figure 4C), at baseline (Figure S7B, Supporting Information) and, for studies monitoring cells also in untreated disease, those that define Tetramer^+^ cells in untreated CeD combined with d6 (Figure 4D). In all cases, integrin *β*7 turned out as the single best prediction marker (Tables S6–S8, Supporting Information). Strikingly, CD95 was among the top 8 markers to define Tetramer^+^ at BL. We suggest that the top 8 markers listed for the three different conditions should be used to monitor pathogenic CD4^+^ T cells in CeD and potentially also in other T‐cell driven diseases. For diseases not associated directly to the gut, the gut‐homing markers may also be substituted according to the correlation analysis in Figure 4B.

### 2.7 Parallels between CeD‐Associated CD8^+^ and *γδ* ^+^ T Cells and Gluten‐Specific CD4^+^ T Cells

**Figure 5:**
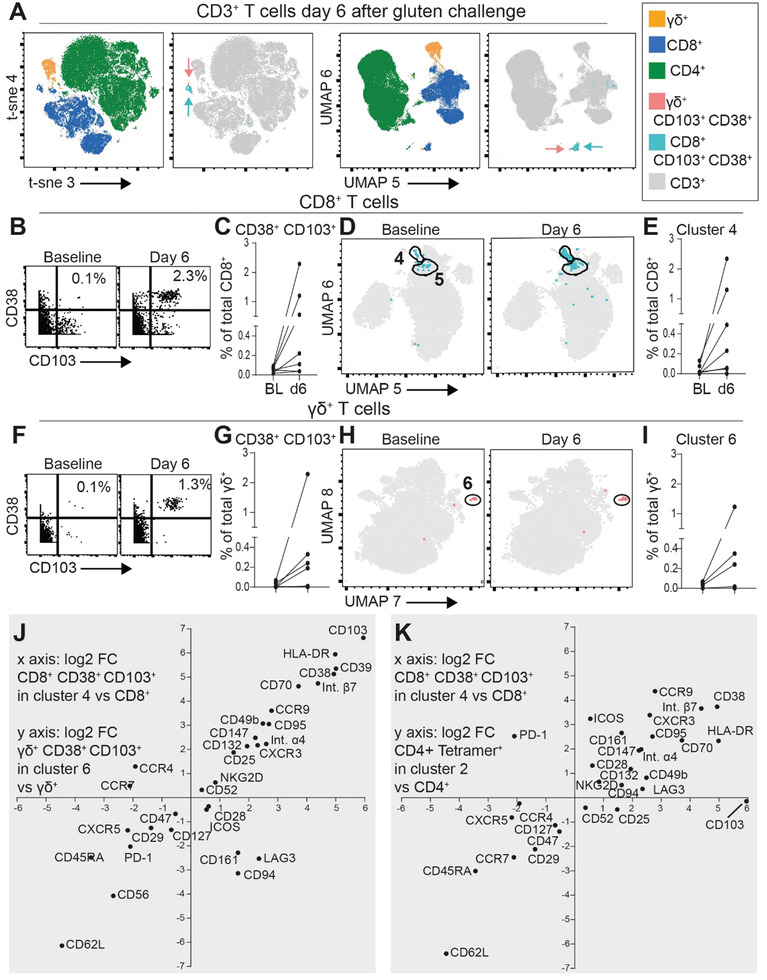
Comparing phenotypic characteristics of celiac disease‐associated (CD38^+^, CD103^+^) CD8^+^ and *γδ* ^+^ T cells with gluten‐specific CD4^+^ T cells at day 6 after gluten challenge. A) Mass cytometry‐derived t‐sne and UMAP plots depicting all CD3^+^ T cells, with CD8^+^, *γδ* ^+^, and CD4^+^ T‐cell types in addition to the CD38^+^ CD103^+^ subsets of CD8^+^ (turquoise arrows) and *γδ* ^+^ (red arrows) T cells color coded, on day 6 after gluten challenge. B) Percentage of CD103^+^ CD38^+^ cells among CD8^+^ T cells in a representative CeD patient C) and in *n* = 7 CeD patients on baseline and day 6. D) UMAP plot highlighting CD103^+^ CD38^+^ cells among CD8^+^ T cells, E) with percentage of cells within cluster 4 at baseline and day 6 (*n* = 7). F) Percentage of CD103^+^ CD38^+^ cells among *γδ* ^+^ T cells in a representative CeD patient G) and in *n* = 7 CeD patients on baseline and day 6. H) UMAP plot highlighting CD103^+^ CD38^+^ cells among *γδ* ^+^ T cells, I) with percentage of cells within cluster 6 at baseline and day 6 (*n* = 7). J) Correlation between mass cytometry‐derived log_2_ fold‐change expression of indicated markers on cluster 4 cells versus all CD8^+^ T cells (*x* axis, *n* = 6, including only those with increase in (C) and cluster 6 cells versus all *γδ* ^+^ T cells (*y* axis, *n* = 4, including only those with detectable increase in (G). K) Correlation between log_2_ fold‐change expression of indicated markers on cluster 4 versus all CD8^+^ T cells (*x* axis) and HLA‐DQ2.5:gluten tetramer‐positive gut‐homing effector memory cells in cluster 2 (see Figure 2) versus all CD4^+^ T cells (*y* axis, *n* = 6). The median log_2_ fold changes from the CeD patients analyzed on day 6 of oral gluten challenge are depicted J,K).

A large number of the highly correlated markers expressed by gluten‐specific CD4^+^ T cells on day 6 (Figure 4B), are associated with T‐cell activation and gut homing. As gluten challenge‐associated (CD38^+^ CD103^+^) CD8^+^ and *γδ* ^+^ T cells overlap clonally with CD8^+^ and *γδ* ^+^ T cells in the celiac lesion,^[^ ^15^ ^]^ we asked if they would express an overlapping pattern of activation and gut homing markers (modified mass cytometry staining panel in Table S9 in the Supporting Information and gating strategy in Figure S9 in the Supporting Information). For this analysis, we utilized the tetramer‐depleted fraction of the PBMCs and initially analyzed all CD3^+^ T cells together. Strikingly, the CD38^+^ CD103^+^ CD8^+^ and *γδ* ^+^ cells clustered together and apart from all other CD3^+^ T cells in the high‐dimensional t‐sne and UMAP analyses (**Figure** **5**A). The clustering indicated that there is high correlation between the phenotype of these particular CD8^+^ and *γδ* ^+^ T cells and that they have a very distinct phenotype. Focusing on the CD8^+^ T cells, we observed an increase in CD38^+^ CD103^+^ cells in five of six the CeD patients analyzed (Figure 5B,C). The CeD‐associated CD8^+^ and *γδ* ^+^ T cells were selected already on the basis of having an activated phenotype (i.e., CD38^+^). Thus, by contrast to Tetramer^+^, these cells located within the same two clusters at baseline and at d6, here termed cluster 4 and cluster 5. Notably, an increased frequency was only detectable within cluster 4 (Figure 5E; Figure S8A, Supporting Information). For the CD38^+^ CD103^+^ *γδ* ^+^ T cells, we detected an increase in four of the six gluten‐challenged CeD patients (Figure 5F,G). Similar to the CD38^+^ CD103^+^ CD8^+^ T cells, but again contrasting gluten‐specific CD4^+^ T cells, the CD38^+^ CD103^+^ *γδ* ^+^ T cells did not display dramatic phenotypic changes upon gluten challenge. These *γδ* ^+^ T cells located within one and the same cluster, here termed cluster 6, and cells within this cluster increased upon gluten challenge (Figure 5H,I). Furthermore, and in accordance with the close proximity of CD38^+^ CD103^+^ CD8^+^ and *γδ* ^+^ T cells in the UMAP‐plot including all CD3^+^ T cells (Figure 5A), we found high degree of correlation between the herein included in the mass cytometry staining markers between these CeD‐associated CD8^+^ and *γδ* ^+^ T cells (Figure 5J). Finally, we also observed high correlation between the activation‐ and homing markers expression by the gluten‐specific CD4^+^ T cells and the CD38^+^ CD103^+^ CD8^+^ T cells mobilized into blood on day 6 after oral gluten challenge (Figure 5K). Here, PD‐1 (highly expressed on Tetramer^+^ cells) and CD103 were two noticeable exceptions that did not correlate well between these two cell types.

## 3 Discussion

Among HLA‐class II‐associated disorders, CeD offers a unique possibility to study disease‐driving, pathogenic CD4^+^ T cells, given the knowledge of the disease‐driving antigen and the availability of HLA‐DQ:gluten tetramer tools.^[^ ^26^, ^27^ ^]^ In this study, we have characterized HLA‐DQ:gluten tetramer^+^ cells with RNA‐seq and mass cytometry and demonstrate how the phenotype of the cells shifts as a function of gluten exposure. We define a distinct set of markers that can be used to identify pathogenic T cells in CeD and which can be used for cell monitoring and for development of drugs that target the culprit T cells.

We report that gluten‐specific CD4^+^ T cells have a distinct phenotype in gluten challenged and treated CeD patients. As previously shown, such cells also have a narrow phenotype in gut and blood of untreated CeD patients.^[^ ^6^ ^]^ Together, these findings indicate that gluten‐specific CD4^+^ T cells can be therapeutically targeted within different disease states. However, our data also indicate that they are a “moving therapeutic target,” shifting in expression of some phenotypic markers with ingestion of gluten (Figure 2). Drugs aiming at checkpoint molecules or activation markers may thus have very limited therapeutic time windows. On the other hand, the stable expression of markers associated with gut homing or inflamed tissues (integrin *α*4*β*7, CCR9, and CXCR3) (Figures 2 and 3), indicates that blocking such molecules may be successful when treating CeD over longer period of time.^[^ ^12^ ^]^ Nonetheless, it is possible that similarly dynamic shifts in phenotypic features of pathogenic T cells will play out in other autoimmune conditions as well, especially those including flares or other fluctuating courses of disease activity. Thus, our observations are likely relevant for therapies beyond CeD.

Which antigen(s) the CeD‐associated CD8^+^ and *γδ* ^+^ T cells that increase on day 6 after gluten challenge, are specific for, remain unknown. Thus, we cannot visualize these cells with an antigen‐specific “flag”; rather they are identified by their activated (CD38^+^) and gut‐homing profile (CD103^+^). The lack of shift in profile of these cells between day 0 and day 6 of the gluten challenge may relate to the cells being selected on basis of having an activated phenotype. The overlap of activation‐ and gut homing markers between the CeD‐associated CD8^+^ and *γδ* ^+^ T cells and the gluten‐specific CD4^+^ T cells increasing in blood on day 6, is notwithstanding striking. It may suggest that all these cell subsets can be targeted for therapeutic purposes though common surface‐expressed markers, such as CXCR3, integrin *α*4*β*7, CCR9, CD70, and CD147. Here, it is worth mentioning that we, despite a potential pathogenic role via secretion of IFN‐*γ*,^[^ ^28^ ^]^ know very little about the role of the intraepithelial and circulating CD38^+^ CD103^+^ *γδ* ^+^ T cells in CeD. The effects of depletion of such cells through targeted immunotherapy is hence difficult to foresee.

The markers reported in Figure 4B,D can be helpful when selecting a simple flow staining panel to monitor activated gluten‐specific CD4^+^ T cells. Moreover, multiple studies from the last three years have shown that CD4^+^ T cells, phenotypically highly similar to gluten‐specific CD4^+^ T cells, are increased also in other autoimmune conditions.^[^ ^29^, ^30^, ^31^ ^]^ Furthermore, and similar to what is observed for CD38^+^ CD103^+^ CD8^+^ and *γδ* ^+^ T cells in CeD^[^ ^13^ ^]^ CD38^+^ CD8^+^ and *γδ* ^+^ T cells homing to the brain in patients with newly diagnosed multiple sclerosis show massive oligoclonal expansion.^[^ ^13^, ^32^ ^]^ Thus, the high correlation of markers expressed by gluten‐specific CD4^+^ T cells (Figure 4B) and the overlapping phenotypic features of CeD‐associated CD8^+^ and *γδ* ^+^ T cells, suggests that several, almost equally well performing markers can be used for monitoring disease‐relevant cells in CeD and likely other autoimmune conditions.

Gluten‐specific CD4^+^ T cells seem to be capable of entering the small intestine continuously (high, stable integrin *α*4*β*7/CCR9/CXCR3 expression). Nonetheless, the unique CXCR6‐expression of gluten‐specific CD4^+^ on d6, indicates that the CXCR6/CXCL16 axis is involved in the increased migration of such cells into the gut with the ingestion of gluten. Indeed, we have previously demonstrated that that the number of gluten‐specific cells increases in the small intestine with oral gluten challenge,^[^ ^7^ ^]^ and by global RNA analysis of gut biopsies it was demonstrated that CXCR6 is increased in CeD patients compared to controls.^[^ ^33^ ^]^ The CXCR6/CXCL16 axis is important to the recruitment of tissue‐resident CD8^+^ memory T cells in the airways,^[^ ^34^ ^]^ and in mouse models, to the positioning of disease‐specific T cells within the inflamed tissue.^[^ ^35^, ^36^ ^]^ Notably, CXCR6 was not included in the panel staining CD8^+^ and *γδ* ^+^ T cells. Nonetheless, we would argue that the CXCR6/CXCL16 axis is important to the recruitment newly activated gluten‐specific CD4^+^ T cells to the gut, and that the gluten‐specific CD4^+^ T cells peaking on d6 after challenge become tissue resident. Interestingly, the serum levels of CXCL16 reflect disease activity in multiple sclerosis, juvenile systemic lupus erythematosus and systemic sclerosis^[^ ^37^, ^38^, ^39^ ^]^ and CXCL16 is upregulated with intestinal inflammation such as Crohn's disease.^[^ ^40^ ^]^ Together, these observations imply that the CXCL16/CXCR6 axis can be a therapeutic target in CeD and other autoimmune conditions.

Interestingly, some gluten‐specific CD4^+^ cells at baseline had increased CD95 expression (Figure S4 in the Supporting Information and listed as top eight predictors at baseline in Table S8 in the Supporting Information). The implications of CD95 expression by these cells is currently unclear. CD95 (Fas) by interaction with CD95L is involved in apoptosis, but recent data have revealed that CD95 also evokes nonapoptotic signals and promotes inflammation.^[^ ^41^ ^]^

Understanding where and how gluten‐specific T cells are activated is obviously important to be able to fully eradicate such cells. In untreated CeD patients, we have previously reported that the majority of gluten‐specific T cells lack CXCR5 despite expression of the follicular T (T_FH_) markers PD‐1, CXCL13, and IL‐21. Very similar T cells are also increased in other autoimmune conditions,^[^ ^29^, ^42^ ^]^ allowing us and others to speculate that inductive T‐B‐cell interactions in autoimmune diseases take place outside of germinal centers. As we now show that a subset of gluten‐specific CD4^+^ T cells at baseline express CXCR5 (cluster 1B) and home to lymphoid structures (increased CCR7 expression) in the small intestine (CCR9^+^, integrin *α*4*β*7^+^, and CXCR3^+^), the antigen‐induced initial T‐B‐cell interaction may very well be CXCR5 dependent. In parallel, CXCR5^−^ gluten‐specific T cells and the CD38^+^ CD103^+^ CD8^+^ and *γδ* ^+^ T cells, also lacking CXCR5 expression, may be effector cells migrating to nonlymphoid structures of the small intestine. A few Tetramer^+^ cells also express CXCR5 in untreated CeD, indicating that also during active disease some gluten‐specific CD4^+^ T cells migrate to lymphoid structures in the gut. We have previously not detected CXCR5 on the surface of gluten‐specific cells in the small intestine. Apparently, gluten‐specific CD4^+^ T cells downregulate CXCR5 and stop proliferating (Ki‐67^−^)^[^ ^8^ ^]^ when entering the gut, while still expressing the CXCR5‐ligand CXCL13. Alternatively, CXCR5^+^ gluten‐specific T cells are present in the gut mucosa, but outside the biopsies obtained by gastroduodenoscopy.

Parallel to the upregulation of CXCR6 and the downregulation of CXCR5, markers such as CD132, CD147, CD95, and CD70 were all increased at RNA and cell surface level in gluten‐specific CD4^+^ cells on d6 compared to baseline. CD147, which was also clearly increased in gluten‐specific CD4^+^ cells in untreated CeD (Figure 3) as well as in CD38^+^ CD103^+^ CD8^+^ and *γδ* ^+^ T cells (Figure 5J,K), is widely expressed and have multiple interacting partners.^[^ ^43^ ^]^ When expressed by T cells, it appears to be associated with migration (through interaction with cyclophilin A), proliferation and activation, and it is also increased on T cells in rheumatoid arthritis and other autoimmune conditions.^[^ ^44^, ^45^ ^]^ In addition, plasma levels of CD147 has been reported to reflect the histological disease activity in in patients with lupus nephritis.^[^ ^46^ ^]^ Notably, it was reported that CD147 is important for cyclophilin A‐induced recruitment of CD161^+^ CD4^+^ T cells into synovial fluid in rheumatoid arthritis, and that this recruitment may be reduced by blocking CD147.^[^ ^47^ ^]^ Multiple CD147‐blocking drugs are known. There is hence a rational for testing CD147‐blocking drugs in CeD and other diseases.

The CD70/CD27‐interaction is of major importance for T‐cell co‐stimulation. CD70 is transiently expressed by activated T‐ and B cells, mature dendritic cells^[^ ^48^ ^]^ but also by solid tumors,^[^ ^49^ ^]^ which is why several ongoing trials explore multiple different therapeutic approaches to target CD70 in cancer therapy.^[^ ^50^ ^]^ Interestingly, CD70 is increased in gluten‐specific CD4^+^ T cells in blood of untreated CeD and of treated CeD on d6 after gluten challenge (Figures 2E and 3D), in CD38^+^ CD103^+^ CD8^+^ and *γδ* ^+^ T cells circulating in blood (Figure 5J,K) as well in intraepithelial *γδ* ^+^ T cells of untreated and treated CeD.^[^ ^28^ ^]^ Although the role of CD70 on T cells is not fully understood, it was demonstrated in experimental autoimmune encephalomyelitis disease that CD70 defined a proinflammatory CD4^+^ T‐cell subset, induced by TGF*β*1/3, and that CD27^−/−^ T cells provoked less severe CNS inflammation.^[^ ^51^ ^]^ Furthermore, it was shown that blocking the CD70/CD27‐pathway with anti‐CD27 antibodies, reduces the inflammatory response in arthritis^[^ ^52^ ^]^ and colitis^[^ ^53^ ^]^ in murine experimental models. As with CD147, there is thus a rational for testing therapies targeting CD70. However, as CD70 and CD147 are also expressed by nondisease relevant T cells, bispecific antibodies or similar reagents will be needed specifically target the cells of pathogenic relevance.

In summary, we here report that circulating gluten‐specific T cells in treated CeD on antigen‐exposure induce multiple markers, many which are associated with T‐cell activation, that possibly can be targeted directly with immunotherapy. Furthermore, we report multiple overlapping makers between gluten‐specific CD4^+^ T cells and CeD‐associated CD38^+^ CD103^+^ CD8^+^ and *γδ* ^+^ T cells increasing in blood after ingestion of gluten. Oral gluten challenge of CeD patients should offer a good setting to test drugs interacting with the molecules reported here in a human system.

## 4 Experimental Section

Detailed Experimental Section for the current study is found in the Supporting Information. RNA‐seq raw sequences are available at https://www.ebi.ac.uk/ega under the accession number EGAS00001004988. The source code is available on https://github.com/eivindgl/ced‐challenge‐blood‐cytof. Other data of this study are available from the authors upon reasonable request.

The studies are covered by the regional ethics committee approvals #6544 and #8025. The oral gluten challenge study is registered by ClinicalTrials.gov (NCT02464150). All participants gave informed written consent.

## Conflict of Interest

The authors declare no conflict of interest.

## Supporting information

Supporting Information

Supplemental Table 1

Supplemental Table 2

Supplemental Table 3

Supplemental Table 4

## Data Availability

The data that support the findings of this study are openly available in European Genome‐Phenome Archive at https://ega‐archive.org/, reference number EGAS00001004988.
