# Pathogenic T Cells in Celiac Disease Change Phenotype on Gluten Challenge: Implications for T‐Cell‐Directed Therapies

**DOI:** 10.1002/advs.202205912

**Published:** 2022-12-08

**Authors:** Asbjørn Christophersen, Stephanie Zühlke, Eivind G. Lund, Omri Snir, Shiva Dahal‐Koirala, Louise Fremgaard Risnes, Jørgen Jahnsen, Knut E. A. Lundin, Ludvig M. Sollid

*Adv. Sci*. **2021**, *8*, 2102778

DOI: 10.1002/advs.202102778

In the original published article, there is an error in Figure S1, Supporting Information. Please find the correct Figure S1 below.

**Figure S1:**
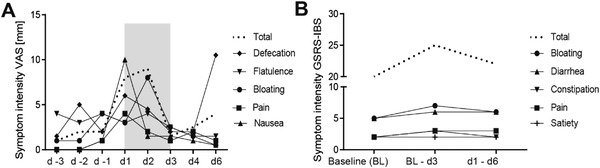
Symptom score. Median of symptoms expressed in a VAS score (maximal value 100 mm) for the categories *Pain, Bloating, Flatulence, Nausea, Satisfaction with defecation* and *Total complaints* and GSRS questionnaire for the categories *Pain, Bloating, Constipation, Diarrhea* and *Satiety* in total n = 17 celiac disease patients undergoing a 3‐day gluten challenge. The patients are listed in Table S1 and Table S4. Grey background indicates time period of gluten ingestion.

The authors apologize for any inconvenience caused.

