# Supplementary material for: Pathogenic T Cells in Celiac Disease Change Phenotype on Gluten Challenge: Implications for T‐Cell‐Directed Therapies
Source: Adv Sci (Weinh). 2021 Sep 8;8(21):2102778. doi: 10.1002/advs.202102778 (PMC8564461; doi:10.1002/advs.202102778)
Supplement: Supplementary file 1 — Supporting Information [file ADVS-8-2102778-s002.pdf]

© 2021 Wiley-VCH GmbH

ADVANCED SCIENCE
Open Access

Supporting Information

for *Adv. Sci.,* DOI: 10.1002/advs.202102778

Pathogenic T cells in Celiac Disease Change Phenotype on Gluten Challenge: Implications for T-cell Directed Therapies

Asbjørn Christophersen, Stephanie Zühlke, Eivind G. Lund, Omri Snir, Shiva Dahal-Koirala, Louise Fremgaard Risnes, Jørgen Jahnsen, Knut E. A. Lundin and Ludvig M. Sollid**

Pathogenic T cells in celiac disease change phenotype on gluten challenge: Implications for T-cell directed therapies

Asbjørn Christophersen, Stephanie Zühlke, Eivind G. Lund, Omri Snir, Shiva Dahal-Koirala, Louise Fremgaard Risnes, Jørgen Jahnsen, Knut E. A. Lundin and Ludvig M. Sollid**

Asbjørn Christophersen, Stephanie Zühlke and Eivind G. Lund contributed equally.
Knut E. A. Lundin and Ludvig M. Sollid contributed equally.
**Corresponding authors: asbjoc@medisin.uio.no, l.m.sollid@medisin.uio.no*

Supporting information

Supplementary Materials and Methods

Supplementary Figures

- Figure S1. Symptom score
- Figure S2. Gating strategy for flow cytometry
- Figure S3. Distribution of markers included in UMAP in Figure 2 and Figure 3
- Figure S4. Comparing phenotypic properties of HLA-DQ2.5:gluten tetramer-binding (Tet^{+}) cells in cluster 1A and 1B
- Figure S5. Correlation between RNA-seq and mass cytometry data from gut homing (integrin$\beta7^{+}$), effector-memory (CD45RA-, CD62L^{-}) CD4^{+} blood T cells
- Figure S6. Localization of cluster 1A and 1B when excluding CXCR5 from the UMAP
- Figure S7. Model performance when integrin β7, CD45RA and CD62L and top 8 markers at baseline
- Figure S8. Percentage of cells within cluster 5 of figure 5D
- Figure S9. Gating strategy for identification of celiac disease-associated (CD103^{+} CD38^{+}) CD8^{+} and $\gamma\delta^{+}$ T cells

Supplementary Tables

- Table S1. Participants for flow cytometry and RNA-seq analysis
- Table S2. Differentially expressed RNA-seq derived genes expressed on the surface of Tetramer^{+} cells
- Table S3. Mass cytometry staining panel for Tetramer$^{+/-}$ CD4^{+} T cells
- Table S4. Participants for mass cytometry analysis
- Tetramer^{+} cells

- Table S5. Untreated celiac disease participants for mass cytometry analysis.
- Table S6. Ten-fold cross validation of the markers that define Tetramer$^+$ cells best at d6 of gluten challenge
- Table S7. Ten-fold cross validation of the markers that define Tetramer$^+$ cells best at d6 of gluten challenge and in untreated CeD
- Table S8. Ten-fold cross validation of the markers that define Tetramer$^+$ cells best at baseline before gluten challenge

Supplementary Materials and methods

Gluten challenge and participants

We first included nine HLA-DQ2.5$^+$ adult CeD patients for gluten challenge and RNA-seq (study design depicted in **Figure 1A**, participants in **Table S1**, Supporting Information). Assessment of material from three additional patients were omitted due to technical issues. All participants had been diagnosed according to the current guidelines[1] and were in clinical and serological remission, indicating that they were well treated on a gluten-free diet.[2] In one participant, IgG-anti-DGP was not completely normalized, despite eight months of adherence to a gluten-free diet (Table S1, Supporting Information). These initial participants were challenged by ingestion of one gluten-containing cookie, containing 8 g of gluten each, three days in a row, as described earlier.[3] For mass cytometry studies of Tetramer$^{+/-}$ CD4$^+$ T cells we included additional six (of seven recruited, as one participant was HLA-DQ2.2$^+$, and thus only included in the studies of CD8$^+$ and $\gamma\delta^+$ T cells summarized in **Figure 5**) individuals (Table S4, Supporting Information) and used residual blood samples from four of the participants recruited for RNA-seq. The seven additional participants recruited for mass cytometry were challenged with four slices of white bread daily, containing approximately 2 g of gluten each, three days in a row, as described earlier.[4] Blood samples were drawn at baseline and d6. Gluten ingestion started on day 1. Finally, we recruited four untreated CeD patients (Table S5, Supporting Information) to compare the phenotype in during gluten challenge and treated CeD patients with previously published phenotype of untreated CeD patients.[5] Healthy controls were not included in this study as we have previously shown that HLA-DQ2.5$^+$ individuals without CeD do not have gluten-specific CD4$^+$ memory T cells detectable with Tetramers.[4, 6]

We measured serum IgA-anti-TG2 (QUANTA Lite R h-tTG IgA ELISA) and IgG-anti-DGP (QUANTA Lite Gliadin IgG II) at the Department of Medical Biochemistry, and typed all

participants for *HLA-DQA1* and *HLA-DQB1* alleles (LABType SSO, ONE LAMBDA) at the Department of Immunology, Oslo University Hospital.

Patient-reported outcome measures

Symptoms were scored daily by a Visual Analogue Scale (VAS) for gastrointestinal symptoms over a period before, during and after the gluten challenge. Scores were obtained for pain, bloating, flatulence, nausea, stool consistency and overall symptoms. We also recorded a modified Gastrointestinal Symptom Rating Scale for patients with irritable bowel syndrome (GSRS-IBS)[7] the last three days at BL, on day 3 and 6 (**Figure S1**, Supporting Information).

HLA-DQ2.5:gluten tetramers

HLA-DQ2.5:gluten tetramers representing five dominant gluten epitopes (DQ2.5-glia-α1a, -α2, -ω1, -ω2 and -hor3) were produced as previously described.[8]

Flow cytometry and RNA sequencing

We isolated PBMCs from fresh whole blood, incubated cells with HLA-DQ2.5:gluten tetramers, enriched for Tetramer-binding cells, while the sample still contained Tetramer$^-$ cells, and stained with an antibody mix as described earlier.[9] $CD3^+/CD11c^-/CD14^-/CD19^-/CD56^-/CD4^+/CD45RA^-/CD62L^-$/integrin $\beta7^+$ cells were separated by sorting (FACS Aria II, BD Biosciences) both as Tetramer$^+$ and Tetramer$^-$ cells (**Figure S2**, Supporting Information) into TCL buffer (Qiagen, Hilden, Germany). Six of the participants with highest number of Tetramer$^+$ integrin $\beta7^+$ T_{EM} cells per 10^6 $CD4^+$ T cells on d6 (Table S1, Supporting Information) were selected for bulk RNA-seq. RNAClean XP magnetic beads (Agencourt Bioscience) were used to extract RNA from samples according to the manufacturer's protocol. We assessed the RNA amount and quality with the RNA 6000 Pico kit on the Bioanalyzer (both Agilent Technologies) and adjusted the RNA concentration to approximately 90 ng/µl before cDNA synthesis (42°C for 90 min; 70°C for 10 min) and amplification (95°C for 1 min; [98°C for 10 sec; 65°C for 30 sec; 68°C for 3 min] for 13 cycles and 72°C for 10 min using the SMART-Seq v4 Ultra Low Input RNA Kit for Sequencing (Takara)). The amplified cDNA was quantified using the High Sensitivity DNA Kit (Agilent Technologies). Tagmentation and adapter ligation were achieved using NexteraXT library preparation kit (Illumina). We sequenced the amplicon libraries on

NextSeq500 (Illumina) at the Norwegian Sequencing Centre (https://www.sequencing.uio.no/).

Mass cytometry staining

For mass cytometry analysis of Tetramer$^{+/-}$ CD4^{+} T cells, we analyzed 10 paired baseline and d6 samples (Table S1 and S4, Supporting Information). We used a novel antibody panel (see data analysis), HLA-DQ2.5:gluten tetramers and our previously established staining protocol.[5, 10] For barcoding, we used anti-CD45 coupled with 89Y, 113In, 106Pd or 110Pd.[11] Paired baseline and d6 samples were thawed and barcoded separately before they were merged and stained in onetube. After enrichment of HLA-DQ2.5:gluten tetramer stained cells, we paired 1 million from the baseline and d6 samples of the already barcoded Tetramer-depleted PBMCs and stained these cells in total 100 µl CyTOF buffer, with a modified mass cytometry staining panel (Table S9, Supporting Information). Although different mass cytometry staining panels for CD4^{+} T cells versus CD8^{+} and γδ^{+} T cells (Table S3 and S9, respectively) we used the same staining approach as previously explained.[5]

Data analysis

Transcript abundance was estimated from fastq files against cDNA for the human reference genome (GRCh38 ensamble 91) using the Kallisto alligner[12] and aggregated at a gene level using tximport.[13] Differentially expressed genes were identified using DESeq2.[14] We adjusted for donor variance and used an adjusted p-value, <0.01. Uncertain log fold change (FC) estimates were reduced using the lfcShrink function (Deseq2 package).[15] We mapped the list of DE genes against a cell surface protein atlas,[16] generating a list of cell-surface expressed proteins on Tetramer^{+} cells (Table S2, Supporting Information), for which we identified 15 corresponding, commercially available antibodies and designed a mass cytometry panel that also contained 16 antibodies (including anti-CD45RA, anti-CD62L and anti-integrin β7) used to characterize gluten-specific CD4^{+} T cells in untreated CeD.[5]

We used FlowJo version 10.6 (FlowJo, LLC) to analyze mass and flow cytometry data and GraphPad Prism 8 (GraphPad Software) for statistical analysis. Mass cytometry-derived pre Tetramer-enriched and Tetramer-enriched samples were gated as previously shown,[5] down-sampled to maximum 100 and 10 000 cells, respectively, before visualization with UMAP and t-sne and extracting mean T-cell marker expressions for visualization (**Figure 2**, **Figure 3, Figure S4**, Supporting Information). The median $\log_2$ FC for expressed T-cell markers were

visualized as heatmaps in *R* with *ggplot* (**Figure 2C-F**), plotted against the corresponding RNA-seq derived median log_2 FC in GraphPad Prism (**Figure S5**, Supporting Information). In parallel, the $CD8^+$ and $\gamma\delta^+$ T cells were down-sampled to maximum 15 000 cells, before visualization with UMAP (**Figure 5D-E, 5H-I**) and extracting mean T-cell marker expressions for each patient. The median log_2 FC for expressed markers on $CD8^+$ T cells ($CD103^+$/$CD38^+$ T cells versus all $CD8^+$) were plotted against that of $\gamma\delta^+$ T cells and versus $Tetramer^+$ $CD4^+$ T cells on day 6, respectively (**Figure 5J, 5K**).

For the ranking and correlation studies visualized in **Figure 4**, we preprocessed and read fcs files in *R* as previously described.[5] The fcs files were exported from FlowJo and pre-gated on $CD4^+$, gut-homing $Tetramer^+$ T_{EM} cells and $CD4^+$ $Tetramer^-$ cells, respectively, and down-sampled to a maximum of 300 cells per sample. For **Figure 4B** we used a standard Pearson correlation analysis with the already balanced down-sampled dataset to identify commonly co-expressed surface proteins in the $Tetramer^+$ population with $Tetramer^-$ cells as a backdrop. For the analysis in **Figure 4C, 4D** we used the *tidymodels framework* in *R* to fit several different classification models, but discovered that a simple logistic regression model was fully able to differentiate the two cell populations. Then, we iteratively fitted logistic regression models on this dataset and used 10-fold cross validation with three repeats at each step to evaluate model performance with ROC-AUC. The weakest predictor with the highest p-value was removed at each step until a single predictor was left.

The untreated CeD and d6 classification in **Figure 4D** was identically performed, except for the addition of the four untreated CeD samples, which were down-sampled to 164 cells/sample (the number of cells in the largest $Tetramer^+$ untreated CeD sample and the subsampling of challenge samples to 40 cells/sample to obtain balance between untreated CeD and challenge samples.

Supplementary Figures

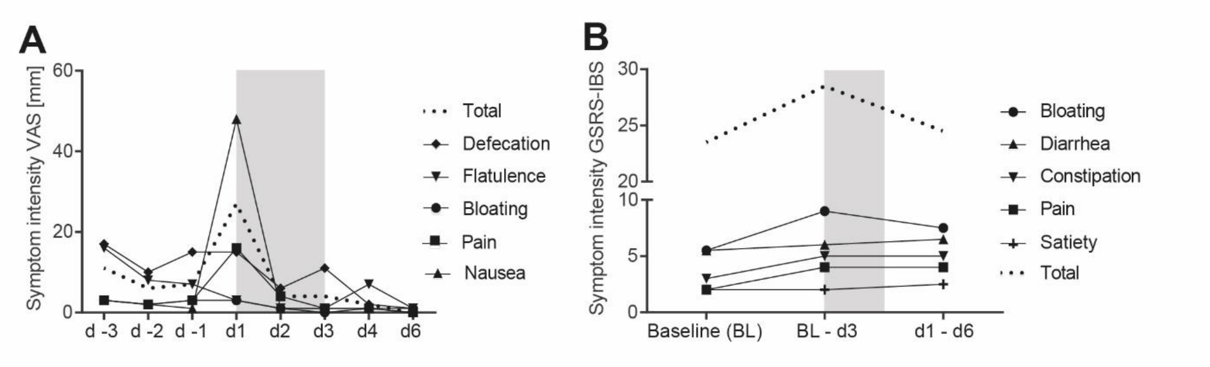

Figure S1. Symptom score

Mean of symptoms expressed in a VAS score (maximal value 100 mm) for the categories *Pain*, *Bloating*, *Flatulence*, *Nausea*, *Satisfaction with defecation* and *Total complaints* and GSRS questionnaire for the categories *Pain*, *Bloating*, *Constipation*, *Diarrhea* and *Satiety*. Grey background indicates time period of gluten ingestion.

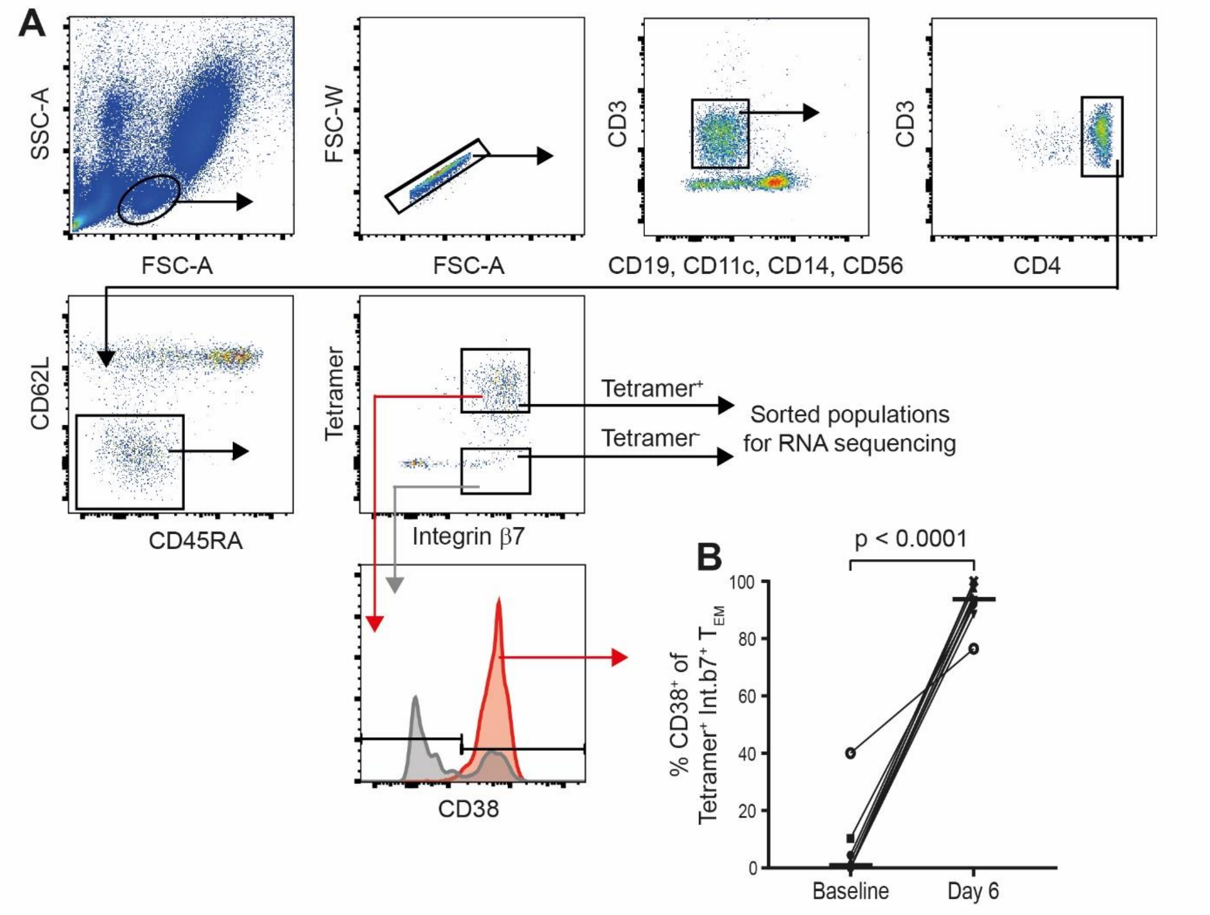

Figure S2. Gating strategy for flow cytometry

A. Gating strategy for sorting HLA-DQ2.5:gluten tetramer^{+}/$^{-}$ effector memory T (T_{EM}) cells from blood after oral gluten challenge. **B.** Increase in CD38 expression on HLA-DQ2.5:gluten tetramer^{+}/$^{-}$ T_{EM} cells in blood on baseline (BL) and 6 days (d6) after oral gluten challenge (paired t-test used to calculate p, median value indicated).

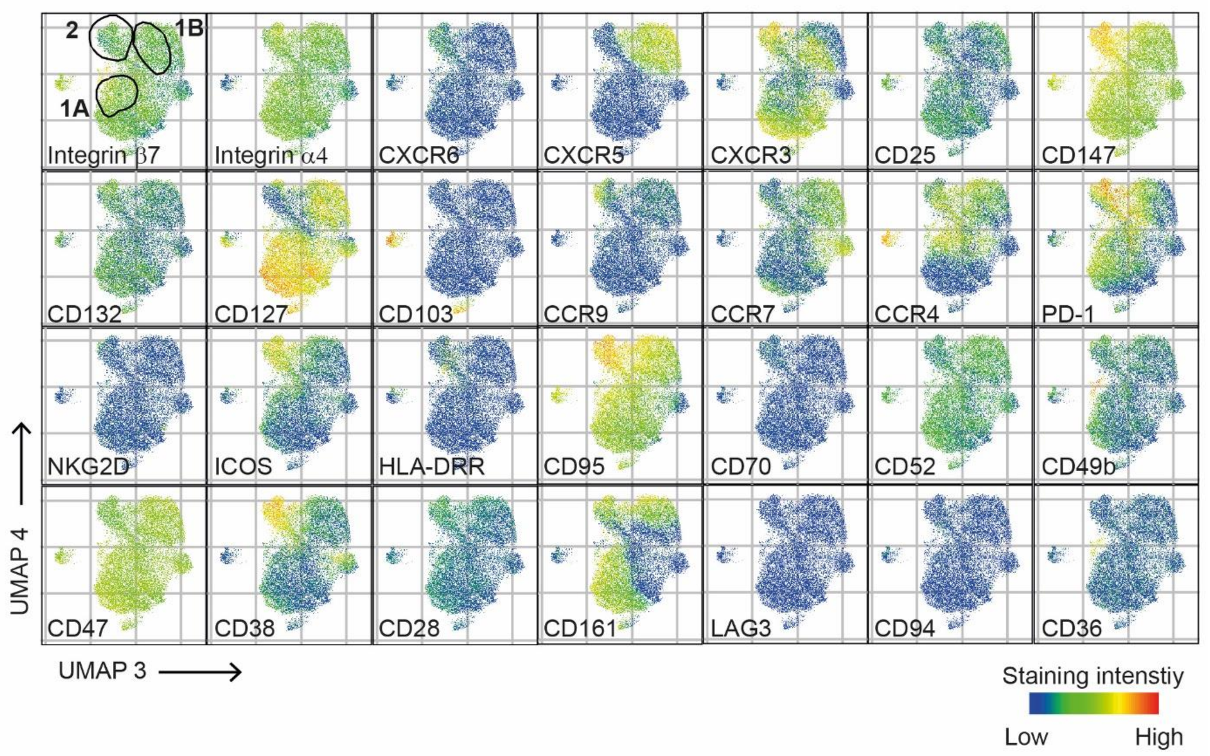

Figure S3. Distribution of markers included in UMAP in Figure 2 and Figure 3

Heat map statistic depicting staining intensities for all indicated markers and integrin $\beta 7^{+}$ T_{EM} cells included in the UMAP plot in Figure 2 and Figure 3. Integrin $\beta 7^{+}$ T_{EM} cells from ten gluten challenge patients at baseline and day 6 and 4 untreated CeD patients were included in the plot.

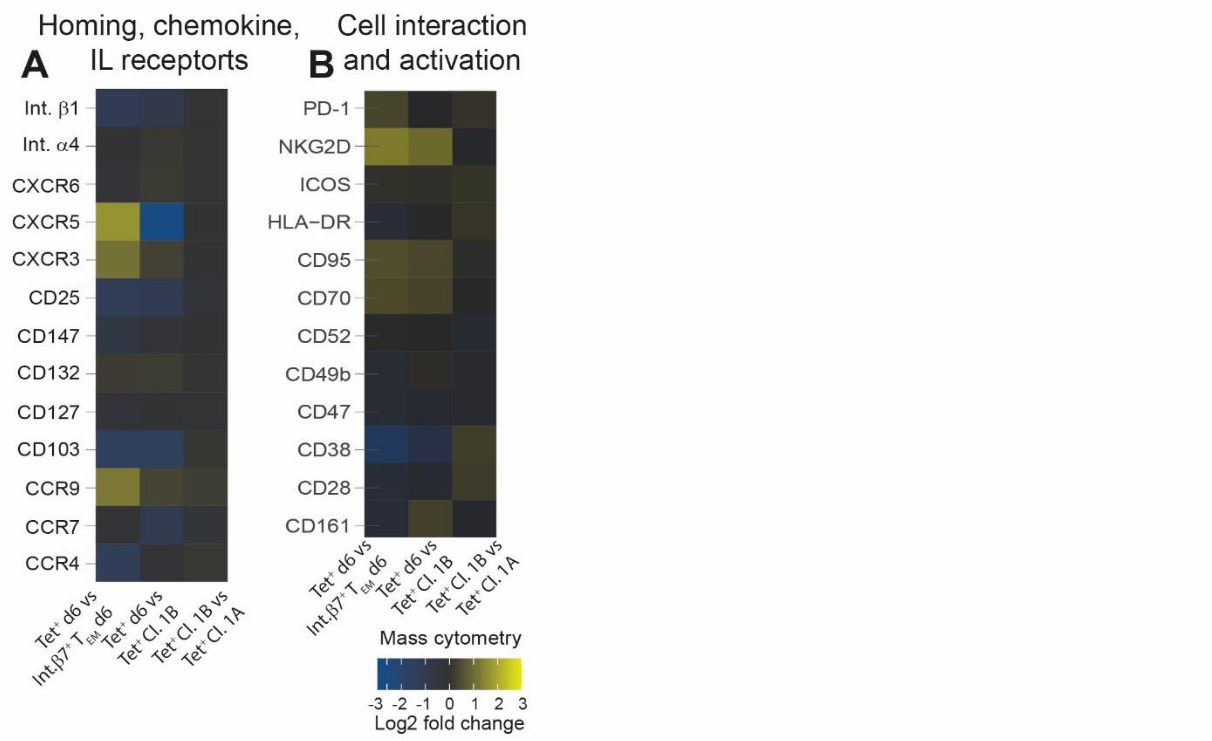

Figure S4. Comparing phenotypic properties of HLA-DQ2.5:gluten tetramer-binding (Tet^+) cells in cluster 1A and 1B

A. Heat map depicting log2 fold change (FC) of indicated homing markers, chemokine and interleukin (IL) receptors **B.** and activation markers analyzed with mass cytometry, comparing Tet^+ cells from cluster 1A, 1B and integrin-$\beta7^+$ T_{EM} cells in general on baseline (BL) and day 6 (d6) as indicated below the heat maps.

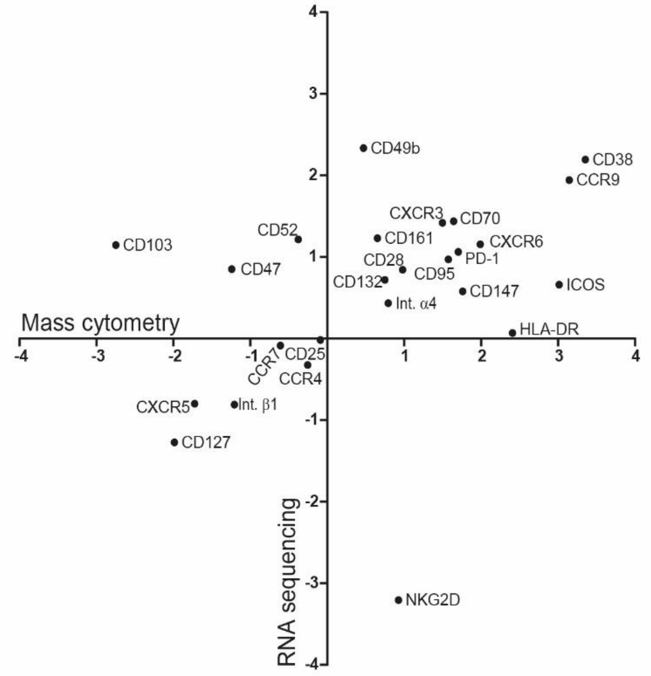

Figure S5. Correlation between RNA-seq and mass cytometry data from gut homing (integrinβ7^{+}), effector-memory (CD45RA-, CD62L^{-}) CD4^{+} blood T cells

Correlation between mass cytometry-derived (*n* = 10) and RNA-seq-derived (*n* = 7) $\log_2$ fold-change expression of indicated markers on HLA-DQ2.5:gluten tetramer-positive versus CD4^{+} gut-homing T_{EM} cells in general. The median $\log_2$ fold changes from celiac disease patients analysed on day 6 of oral gluten challenge are depicted. CD45RA, CD62L and integrin β7 are not included here, as these markers were used for cell sorting prior to RNA-seq.

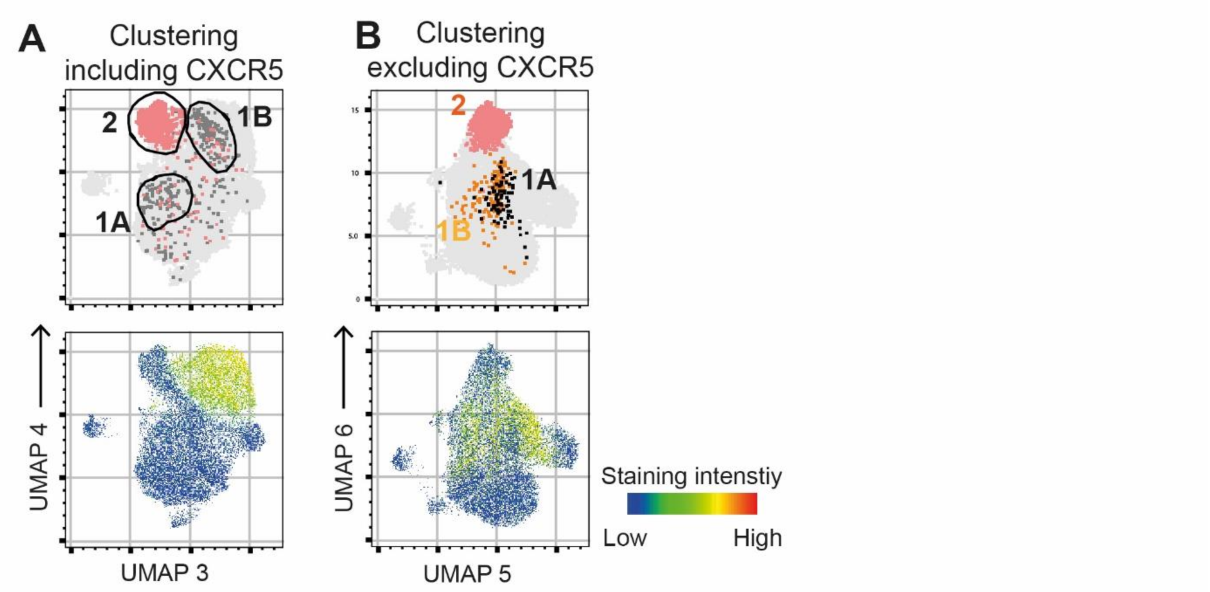

Figure S6. Localization of cluster 1A and 1B when excluding CXCR5 from the UMAP

A. Cluster 1A and 1B (upper plot) differ by the expression of CXCR5, which is highlighted in the lower plot. **B.** A new UMAP plot excluding CXCR5 was made to analyze to which degree cluster 1A and 1B depend on CXCR5 (upper plot). CXCR5 is highlighted in the novel UMAP plot (lower plot).

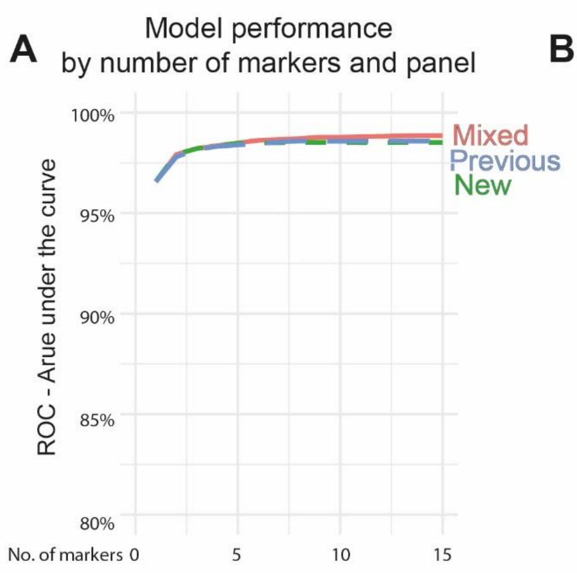

B Identifying gluten-specific cells at baseline

Order	Maker	p value
1	cd47	2E-06
2	int_b7	2E-41
3	cxcr3	1E-07
4	cd95	1E-07
5	nkg2d	4E-05
6	cd62l	2E-10
7	cd29	1E-03
8	cd25	7E-07

Figure S7. Model performance when integrin β7, CD45RA and CD62L and top 8 markers at baseline

A. Comparing three set (panels) of markers when including integrin β7, CD45RA and CD62L. **B.** Top 8 markers to define Tetetramer^{+} at baseline. P values reflect the ability of each indicated marker to predict the Tet^{+} cells.

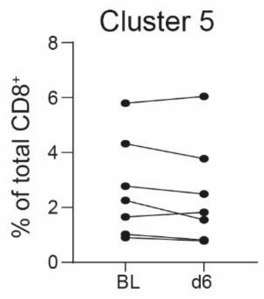

Figure S8. Percentage of cells within cluster 5 of figure 5D. Percentage of cells within cluster 5 of the UMAP plot in Figure 5D highlighting CD103$^+$ CD38$^+$ cells among CD8$^+$ T cells at baseline (BL) and day 6 (d6) (n = 7).

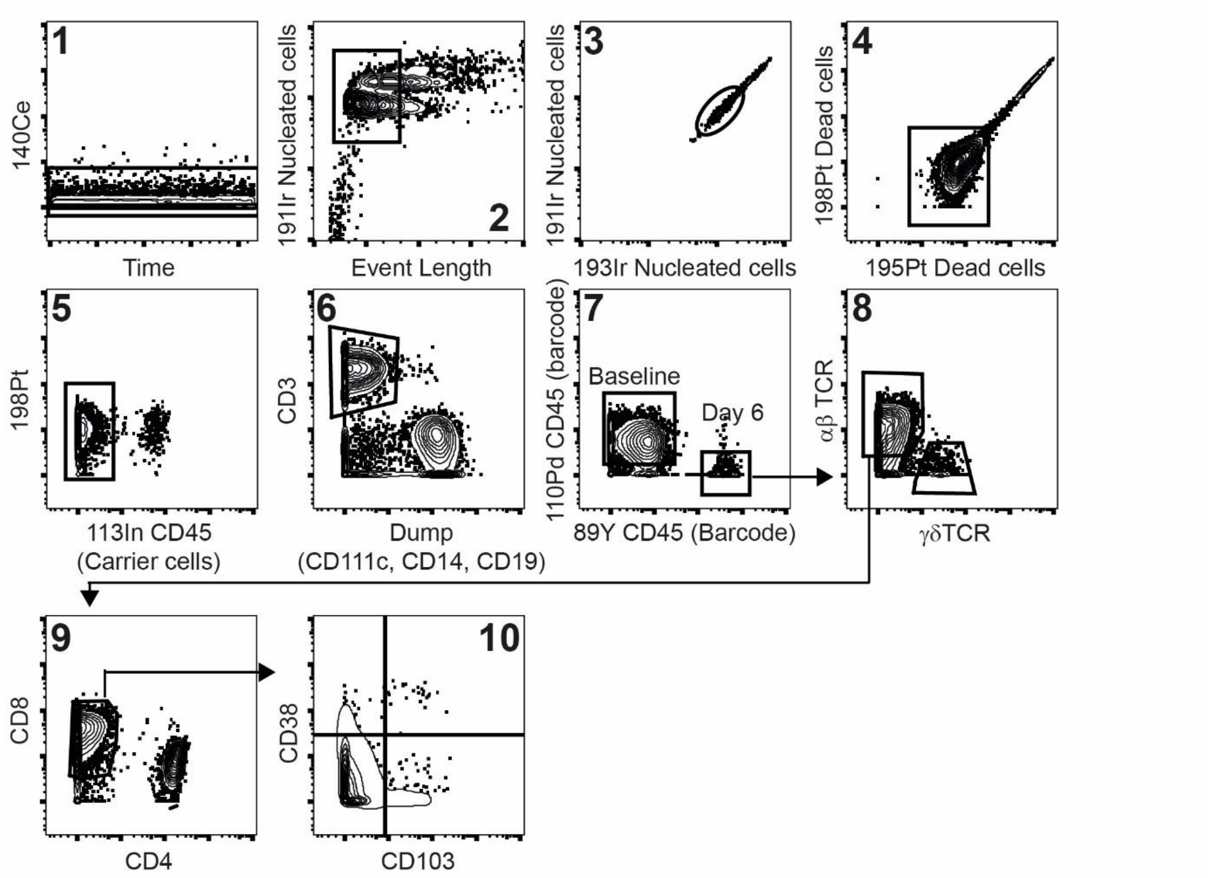

Figure S9. Gating strategy for identification of celiac disease-associated (CD103$^+$ CD38$^+$) CD8$^+$ and γδ$^+$ T cells. Numbers indicate gating order of PBMCs, here exemplified by gating of celiac disease-associated CD8$^+$ T cells on day 6.

Supplementary Tables

Table S1. Participants for flow cytometry and RNA-seq analysis

Individual [a)]	Gender	Age	Years on GFD	anti-TG2 BL [b)]	anti-DGP BL [c)]	Tet^+ / 10^6 $CD4^+$ BL [d)]	Tet^+ / 10^6 $CD4^+$ d6 [e)]	Fold change (d6/BL)
CD1536	M	36	32	<1	<5	17,2	440,8	25,6
CD1454	F	45	0,7	01.apr	29	5,1	119,1	23,2
CD1300	F	62	28	<1	<5	3,6	81,5	22,8
CD1568 [f)]	F	53	7	<1	<5	14,5	53,9	3,7
CD1571	F	54	5	<1	7	0,5	53	104,9
CD1299 [f)]	F	24	11	<1	<5	4,2	47	11,1
CD1582 [f)]	F	55	3,5	<1	<5	1,2	37,7	31,7
CD1575	F	56	13	<1	<5	0,8	7,1	9,3
CD1535	F	24	5	<1	<5	1	3,2	3,2
CD1577 [f) g)]	F	36	9	not done	not done	not done	not done	not done

[a)] The participants were challenged with a gluten-containing cookie. Samples from the participants with the highest absolute number of circulating Tet^+ integrin $\beta 7^+$ T_{EM} cells on day 6 were chosen for RNA-seq. [b)] IgA-anti-TG2, Immunoglobulin A-anti-Transglutaminase 2, Reference value < 4, [AU], arbitrary units. [c)] IgG-anti-DGP, Immunoglobulin G-anti-Deamidated Gliadin Peptide, [AU] Reference value < 20. [d)] Tetramer+ (Tet^+) integrin β7+ T_{EM} cells per 10^6 $CD4^+$ T cells at baseline (BL). [e)] Tetramer+ (Tet^+) integrin β7+ T_{EM} cells per 10^6 $CD4^+$ T cells at day 6 (d6). [f)] Residual blood samples were also used for mass cytometry analysis. Initially recruited for RNA seq and flow cytometry studies, but blood samples only successfully analyzed in mass cytometry studies.

Table S2. Differentially expressed RNA-seq derived genes expressed on the surface of Tetramer$^+$ cells

List of differentially expressed genes (ensembl gene ID and gene name), log2 fold change (lfc) and adjusted p value (padj) for genes that code for proteins expressed on the cell surface as mapped against an atlas of cell-surface proteins.[16]

Table S3. Mass cytometry staining panel for Tetramer$^{+/-}$ CD4^{+} T cells

Label	Target	Clone	Catalog	Concentration [a)]
89Y	CD45	HI30	Fluidigm	1:100
110Pd	CD45	HI30	Biolegend	10 μg/ml
113In	CD45	HI30	Biolegend	8 μg/ml
115In	CD52 [b)]	HI186	Biolegend	1 μg/ml
139La	CD28	CD28.2	Biolegend	7 μg/ml
141Pr	CD49d	9F10	Fluidigm	1:100
142Nd	CD70 [b)]	113-16	Biolegend	8 μg/ml
143Nd	CD278/ICOS	C398.4A	Fluidigm	1:100
144Nd	CD38	HIT2	Fluidigm	1:100
145Nd	CD4	RPA-T4	Fluidigm	0.5:100
146Nd	CD8a	RPA-T8	Fluidigm	0.8:100
147Sm	CD103 [b)]	Ber-ACT8	Biolegend	4 μg/ml
148Nd	CD132 [b)]	TUGh4	Fluidigm	2:100
149Sm	CD56 (NCAM)	NCAM16.2	Fluidigm	0.4:100
150Nd	CD127	A019D5	Biolegend	2 μg/ml
151Eu	CD11c	Bu15	Biolegend	1 μg/ml
151Eu	CD19	HIB19	Biolegend	3 μg/ml
151Eu	CD14	M5E2	Fluidigm	1:100
152Sm	CD36 [b)]	5-271	Fluidigm	1:100
153Eu	CD62L	DREG-56	Fluidigm	0.5:100
154Sm	CD3	UCHT1	Fluidigm	0.8:100
155Gd	CD279 (PD-1)	EH12.2H7	Fluidigm	2.2:100
156Gd	CD29 [b)]	TS2/16	Fluidigm	1:100
158Gd	CD194 (CCR4)	205410	Fluidigm	0.5:100
159Tb	CD161	HP-3G10	Fluidigm	0.5:100
160Gd	CXCR6 (CD186)	K041E5	Fluidigm	2.5:100
161Dy	CD49b [b)]	P1E6-C5	Fluidigm	1.5:100
162Dy	Integrin β7	FIB504	Fluidigm	0.5:100
163Dy	CD183 (CXCR3)	G025H7	Fluidigm	0.8:100
164Dy	CD95 (Fas) [b)]	3164008B	Biolegend	0.6 μg/ml
165Ho	Phycoerythrin	PE001	Fluidigm	0.8:100
166Er	CD314 (NKG2D) [b)]	ON72	Fluidigm	2:100
167Er	CD197 (CCR7)	G043H7	Fluidigm	0.8:100
168Er	CD147 [b)]	306202	Biolegend	1.2:100
169Tm	CD25 (IL-2R)	2A3	Fluidigm	0.6:100
170Er	CD45RA	HI100	Fluidigm	0.8:100
171Yb	CD185 (CXCR5)	RF8B2	Fluidigm	0.75:100
172Yb	CCR9 [b)]	248621	R&D Systems	4:100
173Yb	HLA-DR	L243	Fluidigm	0.75:100
174Yb	CD94 [b)]	HP-3D9	Fluidigm	3:100

175Lu	CD123/LAG3 [b)]	11C3C65	Fluidigm	3:100
176Yb	Allophycocyanin	APC003	Fluidigm	1.25:100
191Ir/193Ir	Nucleateed cells		Fluidigm	
195Pt	Dead cells		Fluidigm	
209Bi	CD47 [b)]	CC2C6	Fluidigm	0.5:100

The panel includes metal tags for sample barcoding (anti-CD45), secondary anti-phycoerythrin staining for identification of HLA-DQ2.5:gluten tetramer-binding cells, secondary anti-allophycocyanin staining for exclusion of HLA-DQ2.5:CLIP2 tetramer binding cells that represent unspecific tetramer binding, viability staining (195Pt) and nucleated cell staining (191/193Ir). [a)] Final concentrations are stated in µg/ml when using self-conjugated antibodies or per volume 100 when the concentration was not available from the manufacturer. [b)] Novel markers based on RNA-seq analysis that were included in the mass cytometry analysis.

Table S4. Participants for mass cytometry analysis

Individual [a]	Gender	Age	Years on GFD	anti-TG2 BL [b]	anti-DGP BL [c]	Tet^+ / 10^6 $CD4^+$ BL [d]	Tet^+ / 10^6 $CD4^+$ d6 [e]	Fold change (d6/BL)
CD2193	M	45	6	1,2	< 5	35,1	1588,2	45,3
CD2192	F	55	13	< 1	< 5	14,7	1446,6	98,5
CD431 [f]	F	49	23	< 1	< 5	159,7	525,6	3,3
CD2206	F	34	7	< 1	< 5	11,7	166,2	14,2
CD2191	F	33	18	1,5	6	5,6	68,8	12,4
CD2194	F	18	6	< 1	< 5	2,1	16,6	7,8
CD2195	F	34	10	< 1	< 5	not done [g]	dot done [g]	

[a] The participants were challenge with gluten-containing bread. [b] IgA-anti-TG2, Immunoglobulin A-anti-Transglutaminase 2, Reference value < 4, [AU], arbitrary units. [c] IgG-anti-DGP, Immunoglobulin G-anti-Deamidated Gliadin Peptide, [AU] Reference value < 20. [d] Tetramer+ (Tet^+) integrin β7+ T_{EM} cells per 10^6 $CD4^+$ T cells at baseline (BL). [e] Tetramer+ (Tet^+) integrin β7+ T_{EM} cells per 10^6 $CD4^+$ T cells at day 6 (d6). [f] Terminated bread challenge after one day due to nausea/vomiting. [g] Unlike the other included participants, being HLA-DQ2.5$^+$ CeD patients, this participant was an HLA-DQ2.2$^+$ CeD patient and thus only included in the staining of CD8+ and γδ+ T cells, i.e. the data in Figure 5, and not in the studies including HLA-DQ2.5:gluten tetramer staining (Figure 1-4).

Table S5. Untreated celiac disease participants for mass cytometry analysis.

Individual	Gender	Age	Marsh score [a)]	anti-TG2 [b)]	anti-DGP[c)]
CD2048	F	64	3A	11,5	76
CD5023	F	27	3B/C	147	66
CD5052	F	44	3A	5,4	2,1
CD5053	M	70	3A	128	89

[a)] The histological appearance in the small intestine was graded according to Marsh as normal mucosa (Marsh score 0), increased number of intraepithelial lymphocytes (Marsh score 1), crypt hyperplasia and hyperplastic lesion (Marsh score 2) and different degree of villous atrophy (Marsh 3A-C). AU, arbitrary units; F, female; M, male; IgA-anti-TG2, Immunoglobulin A-anti-Transglutaminase 2; IgG-anti-DGP, Immunoglobulin G-anti-Deamidated Gliadin Peptide; Ref, Reference value. [b)] IgA-anti-TG2, Immunoglobulin A-anti-Transglutaminase 2, Reference value < 4, [AU], arbitrary units. [c)] IgG-anti-DGP, Immunoglobulin G-anti-Deamidated Gliadin Peptide, [AU] Reference value < 20.

Table S6. Ten-fold cross validation of the markers that define Tetramer$^+$ cells best at d6 of gluten challenge

The weakest predictor was removed stepwise giving one list for each number of markers from 31 down to 1. Each row indicates a predictor used in the model, including the intercept. The *estimate* column is the maximum likelihood estimate of the log odds ratio for each regression term. The *std.error* column indicates the standard error of the estimated regression term in the previous column. The *statistic* column contains the T-statistic for the hypothesis that the regression term is non-zero. The *p.value* column contains the two-sided p-value associated with the t-statistic.

Table S7. Ten-fold cross validation of the markers that define Tetramer$^+$ cells best at d6 of gluten challenge and in untreated CeD

The weakest predictor was removed stepwise giving one list for each number of markers from 31 down to 1. The *estimate* column is the maximum likelihood estimate of the log odds ratio for each regression term. The *std.error* column indicates the standard error of the estimated regression term in the previous column. The *statistic* column contains the T-statistic for the hypothesis that the regression term is non-zero. The *p.value* column contains the two-sided p-value associated with the t-statistic.

Table S8. Ten-fold cross validation of the markers that define Tetramer$^+$ cells best at baseline before gluten challenge

The weakest predictor was removed stepwise giving one list for each number of markers from 31 down to 1. The *estimate* column is the maximum likelihood estimate of the log odds ratio for each regression term. The *std.error* column indicates the standard error of the estimated regression term in the previous column. The *statistic* column contains the T-statistic for the hypothesis that the regression term is non-zero. The *p.value* column contains the two-sided p-value associated with the t-statistic.

Table S9. Mass cytometry staining panel for Tetramer-depleted CD8^{+} and $\gamma\delta^{+}$ T cells

Label	Target	Clone	Catalog	Concentration [a)]
89Y	CD45	HI30	Fluidigm	1:100
110Pd	CD45	HI30	Biolegend	10 μg/ml
113In	CD45	HI30	Biolegend	8 μg/ml
115In	CD52 [b)]	HI186	Biolegend	1 μg/ml
139La	CD28	CD28.2	Biolegend	7 μg/ml
141Pr	CD49d	9F10	Fluidigm	1:100
142Nd	CD70 [b)]	113-16	Biolegend	8 μg/ml
143Nd	CD278/ICOS	C398.4A	Fluidigm	1:100
144Nd	CD38	HIT2	Fluidigm	1:100
145Nd	CD4	RPA-T4	Fluidigm	0.5:100
146Nd	CD8a	RPA-T8	Fluidigm	0.8:100
147Sm	CD103 [b)]	Ber-ACT8	Biolegend	4 μg/ml
148Nd	CD132 [b)]	TUGh4	Fluidigm	2:100
149Sm	CD56 (NCAM)	NCAM16.2	Fluidigm	0.4:100
150Nd	CD127	A019D5	Biolegend	2 μg/ml
151Eu	CD11c	Bu15	Biolegend	1 μg/ml
151Eu	CD19	HIB19	Biolegend	3 μg/ml
151Eu	CD14	M5E2	Fluidigm	1:100
152Sm	γδ TCR [c)]	11F2	Fluidigm	1:100
153Eu	CD62L	DREG-56	Fluidigm	0.5:100
154Sm	CD3	UCHT1	Fluidigm	0.8:100
155Gd	CD279 (PD-1)	EH12.2H7	Fluidigm	2.2:100
156Gd	CD29 [b)]	TS2/16	Fluidigm	1:100
158Gd	CD194 (CCR4)	205410	Fluidigm	0.5:100
159Tb	CD161	HP-3G10	Fluidigm	0.5:100
160Gd	CD39 [c)]	A1	Fluidigm	1:100
161Dy	CD49b [b)]	P1E6-C5	Fluidigm	1.5:100
162Dy	Integrin β7	FIB504	Fluidigm	0.5:100
163Dy	CD183 (CXCR3)	G025H7	Fluidigm	0.8:100
164Dy	CD95 (Fas) [b)]	3164008B	Biolegend	0.6 μg/ml
165Ho	-	-	-	-
166Er	CD314 (NKG2D) [b)]	ON72	Fluidigm	2:100
167Er	CD197 (CCR7)	G043H7	Fluidigm	0.8:100
168Er	CD147 [b)]	306202	Biolegend	1.2:100
169Tm	CD25 (IL-2R)	2A3	Fluidigm	0.6:100
170Er	CD45RA	HI100	Fluidigm	0.8:100
171Yb	CD185 (CXCR5)	RF8B2	Fluidigm	0.75:100
172Yb	CCR9 [b)]	248621	R&D Systems	4:100
173Yb	HLA-DR	L243	Fluidigm	0.75:100
174Yb	CD94 [b)]	HP-3D9	Fluidigm	3:100

175Lu	CD123/LAG3 [b)]	11C3C65	Fluidigm	3:100
176Yb	αβ TCR [c)]	IP26	Fluidigm	1.5:100
191Ir/193Ir	Nucleateed cells		Fluidigm	
195Pt	Dead cells		Fluidigm	
209Bi	CD47 [b)]	CC2C6	Fluidigm	0.5:100

Panel for staining of tetramer-depleted PBMCs, which was used to study $CD8^+$ and $\gamma\delta^+$ T cells. The panel includes metal tags for sample barcoding (anti-CD45), which was performed prior to tetramer staining (see panel in Supplementary Table S3), viability staining (195Pt) and nucleated cell staining (191/193Ir). [a)] Final concentrations are stated in µg/ml when using self-conjugated antibodies or per volume 100 when the concentration was not available from the manufacturer. [b)] Novel markers based on RNA-seq analysis that were included in the mass cytometry analysis. [C)] Markers that were changed compared to the panel made for $CD4^+$ T cells (see Supplementary Table S3).

References

[1] A Al-Toma, U Volta, R Auricchio, G Castillejo, D S Sanders, C Cellier, C J Mulder,K E A Lundin, *United European Gastroenterol J.* **2019**, *7* (5), 583-613.

[2] J F Ludvigsson, C Ciacci, P H Green, K Kaukinen, I R Korponay-Szabo, K Kurppa, J A Murray, K E A Lundin, M J Maki, A Popp, N R Reilly, A Rodriguez-Herrera, D S Sanders, D Schuppan, S Sleet, J Taavela, K Voorhees, M M Walker,D A Leffler, *Gut.* **2018**, *67* (8), 1410-1424.

[3] S Zuhlke, L F Risnes, S Dahal-Koirala, A Christophersen, L M Sollid,K E Lundin, *United European Gastroenterol J.* **2019**, *7* (10), 1337-1344.

[4] M Raki, L E Fallang, M Brottveit, E Bergseng, H Quarsten, K E Lundin,L M Sollid, *Proc Natl Acad Sci U S A.* **2007**, *104* (8), 2831-6.

[5] A Christophersen, E G Lund, O Snir, E Sola, C Kanduri, S Dahal-Koirala, S Zuhlke, O Molberg, P J Utz, M Rohani-Pichavant, J F Simard, C L Dekker, K E A Lundin, L M Sollid,M M Davis, *Nat Med.* **2019**, *25* (5), 734-737.

[6] A Christophersen, L F Risnes, E Bergseng, K E Lundin, L M Sollid,S W Qiao, *J Immunol.* **2016**, *196* (6), 2819-26.

[7] I K Wiklund, S Fullerton, C J Hawkey, R H Jones, G F Longstreth, E A Mayer, R A Peacock, I K Wilson,J Naesdal, *Scand J Gastroenterol.* **2003**, *38* (9), 947-54.

[8] L M Sollid, J A Tye-Din, S W Qiao, R P Anderson, C Gianfrani,F Koning, *Immunogenetics.* **2020**, *72* (1-2), 85-88.

[9] A Christophersen, M Raki, E Bergseng, K E Lundin, J Jahnsen, L M Sollid,S W Qiao, *United European Gastroenterol J.* **2014**, *2* (4), 268-78.

[10] A Christophersen, *HLA.* **2020**, *95* (3), 169-178.

[11] H E Mei, M D Leipold, A R Schulz, C Chester,H T Maecker, *J Immunol.* **2015**, *194* (4), 2022-31.

[12] N L Bray, H Pimentel, P Melsted,L Pachter, *Nat Biotechnol.* **2016**, *34* (5), 525-7.

[13] C Soneson, M I Love,M D Robinson, *F1000Res.* **2015**, *4*, 1521.

[14] M I Love, W Huber,S Anders, *Genome Biol.* **2014**, *15* (12), 550.

[15] A Zhu, J G Ibrahim,M I Love, *Bioinformatics.* **2019**, *35* (12), 2084-2092.

[16] D Bausch-Fluck, A Hofmann, T Bock, A P Frei, F Cerciello, A Jacobs, H Moest, U Omasits, R L Gundry, C Yoon, R Schiess, A Schmidt, P Mirkowska, A Hartlova, J E Van Eyk, J P Bourquin, R Aebersold, K R Boheler, P Zandstra,B Wollscheid, *PLoS One.* **2015**, *10* (3), e0121314.
